# A Comprehensive Analysis of Interferon Regulatory Factor Expression: Correlation with Immune Cell Infiltration and Patient Prognosis in Endometrial Carcinoma

**DOI:** 10.1155/2022/7948898

**Published:** 2022-08-08

**Authors:** Xiaoqin Lu, Rui Li, Yanqi Ying, Wenyi Zhang

**Affiliations:** The Second Affiliated Hospital of Zhengzhou University, China

## Abstract

As a family of transcription factors, the correlations between expression pattern of nine interferon regulatory factor (IRF) family members, the immune invasion pattern, and the associated patient survival rate in endometrial carcinoma (EC) remain to be elucidated. Based on The Cancer Genome Atlas (TCGA), the expression profiles of the high and low IRF mRNA expression groups were analyzed using R (3.6.3) statistical software. Gene annotation and pathway analyses were performed using Metascape. GSEA was performed using the R package clusterProfiler (3.6.3). The single-sample gene set enrichment analysis (ssGSEA) was used to quantify the relative tumor infiltration levels of immune cell types. Immunohistochemistry data provided by HPA database was used to study the expression of the IRF proteins. Using the GEPIA dataset, the correlation between the expression of IRFs and the tumor stage of EC was analyzed. The correlations between the different IRFs were analyzed using cBioPortal. The expression of IRF2, IRF3, IRF5, IRF6, IRF7, IRF8, and IRF9 was different when comparing EC and normal endometrial samples. IRF2, IRF6, IRF7, and IRF8 were indicated to be potential diagnostic markers for EC. In combination with receiver operating characteristic analysis results, IRF2, IRF6, IRF7, and IRF8 were indicated to be potential diagnostic markers for EC. Levels of individual IRFs were associated with alternate outcomes, with the expression of IRF3 being correlated with the stage of EC and high expression of IRF4 being positively correlated with overall survival (OS); conversely, high expression of IRF5 was negatively correlated with OS. Additionally, high expression levels of both IRF2 and IRF4 were positively correlated with the disease-specific survival rate, and high expression of IRF4 was positively correlated with the progression-free interval. These data suggest a role for IRF2, IRF4, and IRF5 in the prognosis of EC. The expression of IRFs is associated with immune infiltration.

## 1. Introduction

The interferon regulatory factor (IRF) family includes nine members: IRF1, IRF2, IRF3, IRF4, IRF5, IRF6, IRF7, IRF8, and IRF9. As transcription factors, all IRF proteins have a conserved amino-terminal DNA-binding domain (DBD) with a helix-loop-helix structure and a motif containing five tryptophan residues [1, 2]. The DBD recognizes the interferon-stimulated response element (ISRE), a consensus DNA sequence element (A/GNGAAANNGAAACT) [3]. The carboxy-terminal region of the IRFs contains a conserved IRF-association domain (IAD)1 or IAD2, which mediates homodimeric and heterodimeric intramolecular interactions with other IRFs, transcription factors, and/or cofactors [4–6]. IRFs control the transcriptional activation of interferons and interferon-stimulated genes (ISGs), and they are key regulators of the Toll-like receptor and interferon signaling [5]. The activation of IRFs is crucial in many basic signal cascades [7].

As one of the most common malignant tumors in women, particularly common in postmenopausal women, endometrial carcinoma (EC) had 382,069 new cases and 89,929 deaths attributed to it worldwide in 2018. The majority of patients are diagnosed early with EC, and the 5-year survival rate is more than 95%. However, late diagnosis, resulting in regional spread or metastasis, causes the 5-year survival rate to decrease to 68% and 17%, respectively. In EC, distant metastases are commonly observed [8–14].

The variances in the expression levels of the IRFs and their association with clinicopathological features have been reported in part in human EC [15, 16]. To the best of our knowledge, bioinformatic analysis has not been applied to explore the role of IRFs in EC. This study tried to study the differential expression, biological function, tumor immune invasion, and clinical prognostic value of IRF family members in patients with EC using bioinformatics methods. Based on The Cancer Genome Atlas (TCGA) databases, we analyzed the expression levels and mutations of the different IRFs in patients with endometrial carcinoma. We analyzed the correlation between IRFs and prognosis of endometrial carcinoma. And we predicted functions and pathways of IRF expression and associated neighboring genes in patients with endometrial carcinoma. The correlation between IRFs and prognosis of endometrial carcinoma and the correlation between IRF expression and immune cell infiltration were performed, so as to determine the role of these transcription factors in endometrial carcinoma.

## 2. Materials and Methods

### 2.1. Data Acquisition

This study included datasets from TCGA database (https://www.cancer.gov/aboutnci/organization/ccg/research/structural-genomics/tcga). We also analyzed RNA-seq transcriptome data in parallel with the corresponding patient clinical data from EC samples. RNA-seq data from 552 EC tissues and 35 adjacent tissues were downloaded from TCGA database (https://portal.gdc.cancer.gov). Patients with EC were categorized as low or high expression and grouped accordingly, based on the median expression levels of the IRFs.

### 2.2. Analysis of Differentially Expressed Genes (DEGs) in the High and Low IRF Expression Groups in EC Samples

The IRF expression profiles of the high and low IRF mRNA expression groups were analyzed using R version 3.6.3 statistical software. DEGs were identified using the Wilcoxon rank sum test. Parameters |log2Fold Change| > 1.5 and adjusted *P* < 0.001 were used to identify DEGs.

### 2.3. Gene Ontology (GO) Analysis

Gene annotation and pathway analyses were performed to analyze the enrichment of IRFs-related DEGs by process and pathway using Metascape (https://metascape.org) [17, 18]. Only terms with *P* < 0.01, a minimum count of 3, and an enrichment factor > 1.5 were considered as significant.

### 2.4. Gene Set Enrichment Analysis (GSEA)

GSEA is an analytical method that determines whether a defined set of genes shows statistically significant, concordant differences when comparing two phenotypes [19]. To elucidate the significant functional and pathway differences between the high and low IRF expression groups, GSEA was performed using the R package clusterProfiler (3.6.3) [20]. The expression level of IRF mRNA was used as a phenotypic label. Adjusted *P* < 0.05, false discovery rate < 0.25, and normalized enrichment score (NES) > 1 were considered as significant enrichment.

### 2.5. Analysis of Immune Infiltration and Its Correlation with IRF Expression

We used the single-sample gene set enrichment analysis (ssGSEA) method from the GSVA package in R to quantify the relative tumor infiltration levels of immune cell types. In this analysis, we integrated the expression of genes in published signature gene lists [21, 22]. The Wilcoxon rank-sum tests and Pearson correlations were used to evaluate the association between immune cell infiltration and mRNA expression of IRFs. The correlation between IRF mRNA expression levels and the infiltration of immune cells was evaluated using the GSVA package (1.34.0) in R (3.6.3) using EC samples from TCGA database [23].

### 2.6. Prognostic Model Generation and Prediction

The multivariate Cox regression analysis and Akaike's information criterion method were used to determine the optimal prognostic model. Additionally, a nomogram was constructed to predict the prognosis using the rms (6.2–0) and survival (3.2–10) packages in R (3.6.3). Patient samples were stratified into high- and low-risk groups based on the median value of their risk scores. The differences in overall survival (OS), disease-specific survival (DSS), and progression-free interval (PFI) between the high- and low-risk groups were determined using the Kaplan–Meier method with a two-sided log-rank test included in the survminer (0.4.9) and survival (3.2–10) packages in R. Receiver operating characteristic (ROC) curves were constructed to evaluate the accuracy of the prognostic model.

### 2.7. Gene Expression Profiling Interactive Analysis (GEPIA) Dataset

GEPIA is a newly developed interactive web server, based on the analysis of RNA sequencing expression data from 9,736 tumors and 8,587 normal samples from TCGA and the Genotype-Tissue Expression projects, which use a standard processing pipeline. GEPIA provides an array of customizable functions including the ability to analyze differential expression in tumor and normal tissues. It also facilitates profiling according to cancer type or pathological stage, patient survival analysis, similar gene detection, correlation analysis, and dimensionality reduction analysis.

### 2.8. TCGA Data and cBioPortal

TCGA holds both sequencing and pathological data on over 30 different cancers. The EC (TCGA, Provisional) dataset includes data from 548 cases with pathology reports, and this was selected for further analysis of IRFs using cBioPortal (http://www.cbioportal.org/index.do?session_id=5b4c1773498eb8b3d566f7b8). The genomic profiles included mutations, putative copy number alterations (CNAs) from genomic identification of significant targets in cancer (GISTIC), mRNA expression Z scores (RNA-seq v.2 RSEM), and protein expression Z scores (reverse phase protein array (RPPA)). Coexpression and networks were calculated according to cBioPortal's online instructions.

### 2.9. The Human Protein Atlas (HPA)

The HPA database, a free public query resource, provides tissue and cell distribution information for all 24,000 human proteins. The Swedish Knut & Alice Wallenberg Foundation, which founded this database, used antibodies and immunohistochemical techniques to examine the distribution and expression of each protein in 48 human normal tissues, 20 tumor tissues, 47 cell lines, and 12 blood cells. The output from this work comprised of 576 immunohistochemical staining maps, which were interpreted and indexed by professionals. The tissues used were taken from 144 different individuals and 216 tumor samples to ensure that the staining results were fully representative. This large-scale protein research project's purpose is to map the protein positions encoded by expressed genes in human tissues and cells.

### 2.10. Statistical Analyses

The Wilcoxon rank-sum tests and Wilcoxon signed-rank tests were used to analyze the expression of IRFs in nonpaired and paired samples, respectively. The ROC curve was generated to evaluate the diagnostic performance of IRF expression using the pROC package (1.17.0.1). One-way analysis of variance was used to analyze the correlation between clinicopathological features and expression of IRFs. Survival curves were produced using the Kaplan–Meier method, and the differences between groups were assessed using the log-rank test. Risk of death was estimated by univariate and multivariate analyses using the Cox proportional hazard modeling. *P* < 0.05 (two-sided) was considered statistically significant. Statistical analyses were carried out using R (3.6.3) and SPSS (25.0).

## 3. Results

### 3.1. Expression of IRFs in Patients with EC

**Figure 1:**
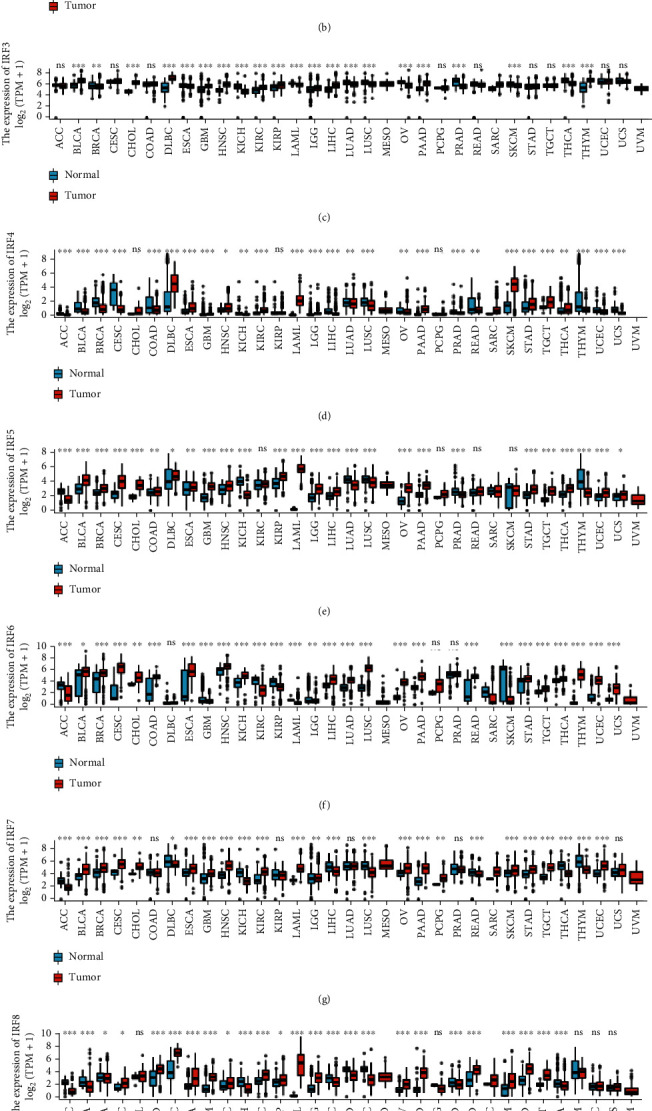
Expression of interferon regulatory factors (IRFs) in different cancers. (a) Expression of IRF1 in different cancers. (b) Expression of IRF2 in different cancers. (c) Expression of IRF3 in different cancers. (d) Expression of IRF4 in different cancers. (e) Expression of IRF5 in different cancers. (f) Expression of IRF6 in different cancers. (g) Expression of IRF7 in different cancers. (h) Expression of IRF8 in different cancers. (i) Expression of IRF9 in different cancers.

Nine IRFs have been identified in mammalian cells. We compared the expression levels of IRFs in a variety of cancer and normal samples using TCGA database (Figure 1).

**Figure 2:**
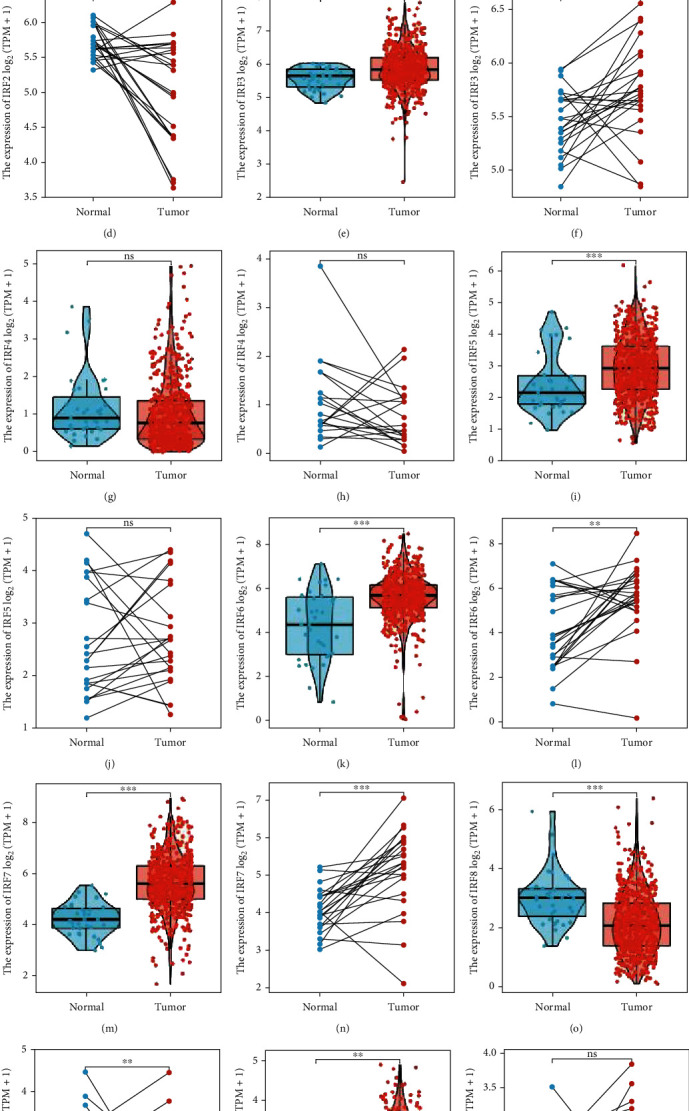
Expression of IRFs in endometrial carcinoma (EC) and normal endometrial samples. (a and b) Expression of IRF1 in paired and unpaired EC and normal endometrial samples, respectively. (c and d) Expression of IRF2 in paired and unpaired EC and normal endometrial samples, respectively. (e and f) Expression of IRF3 in paired and unpaired EC and normal endometrial samples, respectively. (g and h) Expression of IRF4 in paired and unpaired EC and normal endometrial samples, respectively. (i and g) Expression of IRF5 in paired and unpaired EC and normal endometrial samples, respectively. (k and l) Expression of IRF6 in paired and unpaired EC and normal endometrial samples, respectively. (m and n) Expression of IRF7 in paired and unpaired EC and normal endometrial samples, respectively. (o and p) Expression of IRF8 in paired and unpaired EC and normal endometrial samples, respectively. (q and r) Expression of IRF9 in paired and unpaired EC and normal endometrial samples, respectively.

Subsequently, we compared the expression of IRFs in EC and normal endometrium. There were no significant differences in the expression of IRF1 and IRF4 in nonpaired sample tissues. However, variations in the expression levels of the other IRFs were noted, as observed in Figure 2. In paired samples, there was no significant difference in the expression levels of IRF1, IRF4, IRF5, or IRF9 in EC tissues compared to healthy tissue. In similarly paired samples, differences in the expression levels of IRF2, IRF3, IRF6, IRF7, and IRF8 were noted, as shown in Figure 2.

**Figure 3:**
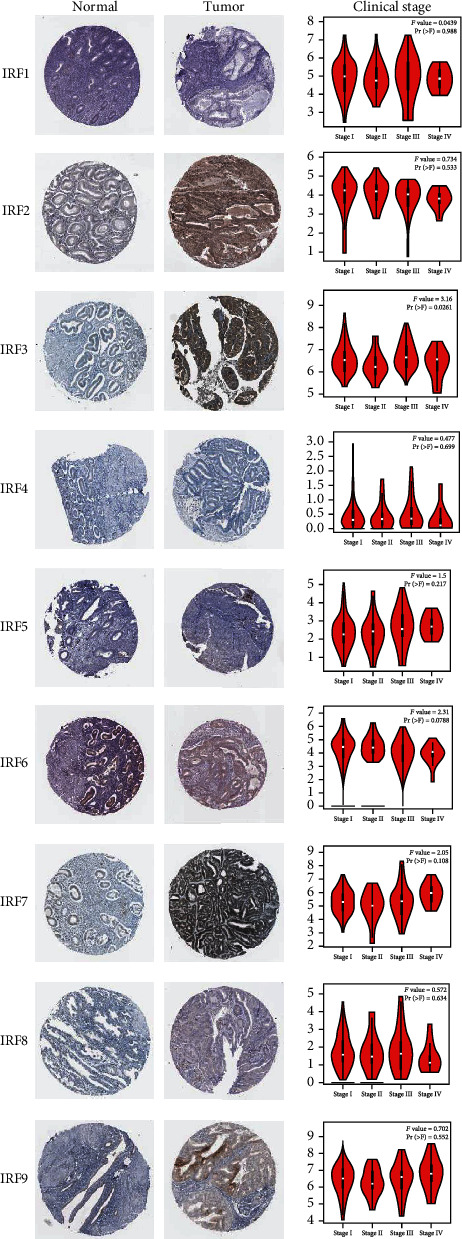
Immunohistochemistry of IRFs from the Human Protein Atlas (HPA) database and the correlation between expression of IRFs and clinical stage of EC (Gene Expression Profiling Interactive Analysis (GEPIA)).

We used the HPA database to determine and study the tissue distribution of the IRF proteins using the immunohistochemistry data provided.  Figure 3 presents the correlation between expression levels of IRFs and clinicopathological parameters in patients with EC.

Using the GEPIA dataset, we analyzed the correlation between the expression of IRFs and the tumor stage of EC. There was a significant difference in the IRF3 group at different tumor stages; however, no difference was observed for any of the other eight IRFs (Figure 3).

### 3.2. Correlation between IRFs and Prognosis of EC

**Figure 4:**
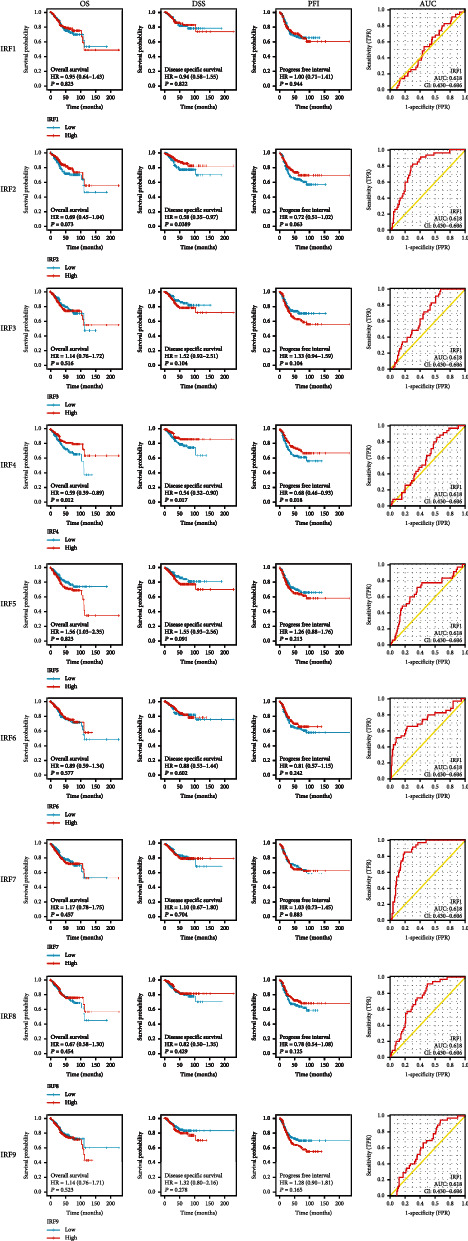
Correlation between overall survival (OS), disease-specific survival (DSS), and progression-free interval (PFI) and expression of interferon regulatory factors (IRFs) in EC and receiver operating characteristic (ROC) analysis of interferon regulatory factors (IRFs) in EC.

Survival analyses, including OS, DSS, and PFI, are shown according to TCGA dataset in Figure 4, respectively.

The high expression level of IRF4 was positively correlated with OS (HR = 0.59, *P* = 0.012), while the high expression of IRF5 was negatively correlated with OS (HR = 1.55, *P* = 0.037). The expression levels of the remaining seven IRFs were OS independent. Individual high expression levels of IRF2 (HR = 0.58, *P* = 0.039) or IRF4 (HR = 0.54, *P* = 0.017) were positively correlated with DSS. The expression levels of the remaining seven IRFs were not correlated with DSS. High expression levels of IRF4 were positively correlated with PFI (HR = 0.66, *P* = 0.018), and the remaining IRFs showed no association with PFI. After plotting the ROC curve (Figure 4), ROC analysis showed that IRF2, IRF6, IRF7, and IRF8 all exhibited positive diagnostic value, with the area under the ROC curve being 0.794, 0.735, 0.877, and 0.736, respectively.

### 3.3. Predicted Functions and Pathways of IRF Expression and Associated Neighboring Genes in Patients with EC

**Figure 5:**
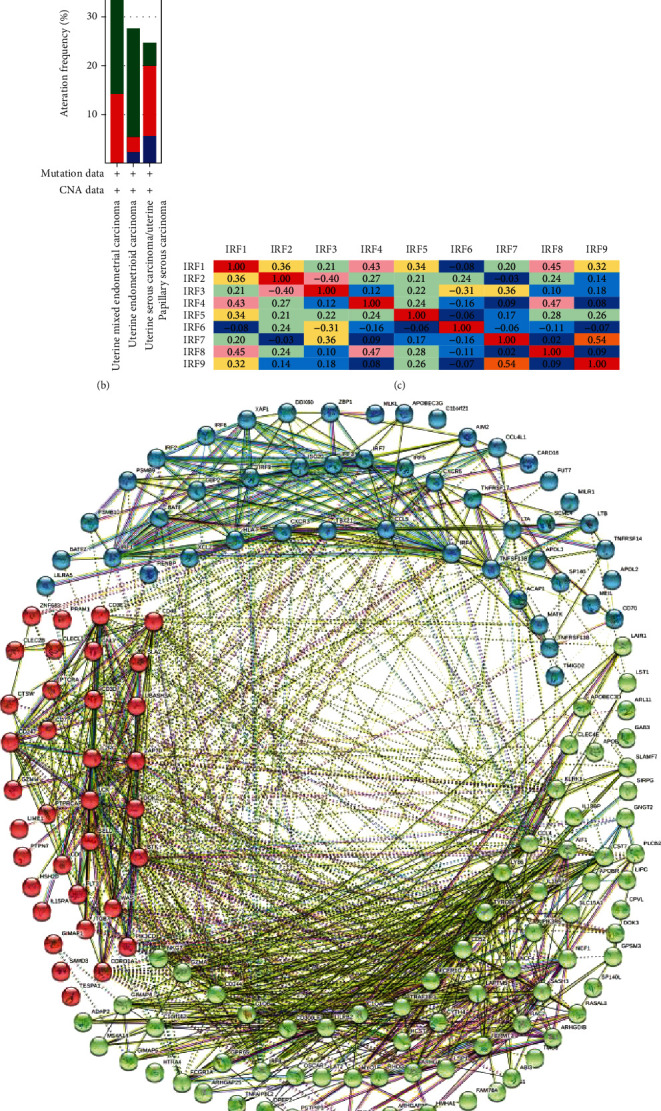
IRF gene expression and mutation analysis in EC (cBioPortal). (a) IRF gene expression and mutation analysis in EC (cBioPortal). (b) Correlation between different IRFs in EC (cBioPortal). (c) Network comprising IRFs and 152 neighboring genes with the most frequently altered expression. (d) Network of IRFs and 152 genes.

We analyzed the variation in IRF expression in 548 samples and evaluated correlations between the different IRFs using cBioPortal EC online tool (TCGA, Provisional; https://www.cbioportal.org/) (Figures 5(a) and 5(b)).

Using the same online tool, mRNA expression was analyzed (RNA sequencing (RNA-seq) version (v.)2 RSEM), and the correlation between IRFs was calculated, with Pearson's correction included. The results showed that IRF1 was positively correlated with IRF2, IRF4, IRF5, IRF8, and IRF9. There was a significant negative correlation between IRF2 and IRF3, and IRF3 was negatively correlated with IRF5 and positively correlated with IRF7. There was a significant positive correlation between IRF4 and IRF8, and a significant positive correlation was noted between IRF7 and IRF9 (Figure 5(c)).

Using the cBioPortal database, we downloaded data about the genes that are coexpressed with IRFs. Taking *P* < 0.001 as the standard, we screened for the genes that were significantly correlated with the expression of IRFs; subsequently, the intersection was taken. Consequently, 152 genes were identified as being significantly closely correlated to the nine IRFs (Supplementary Table 1). We used the STRING database (https://string-db.org/cgi/input.pl) to construct a network for the IRFs and 152 genes (Figure 5(d)). The functions of the IRFs and genes significantly associated with changes in IRF expression were predicted via GO and Kyoto Encyclopedia of Genes and Genomes (KEGG) analyses using Database for Annotation, Visualization, and Integrated Discovery (DAVID) tools (https://david.ncifcrf.gov/summary.jsp).

**Figure 6:**
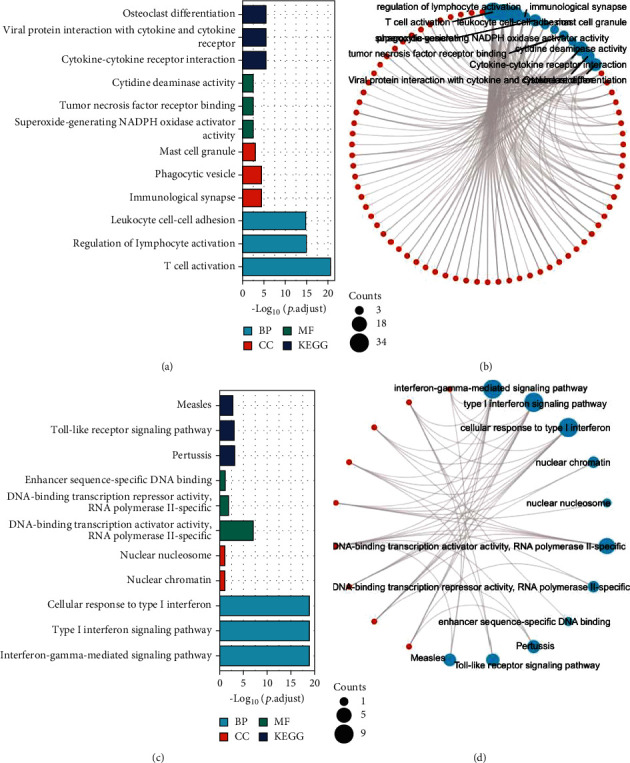
Functions of genes significantly associated with IRF alterations and IRFs. (a and b) GO and KEGG analyses and visualization of genes coexpressed with IRFs. (c and d) GO and KEGG analyses and visualization of IRFs.

GO and KEGG analyses of genes coexpressed with IRFs that were significantly correlated yielded various immunity-associated genes, such as GO:0042110 (T cell activation), GO:0051249 (regulation of lymphocyte activation), GO:0007159 (Leukocyte cell adhesion), GO:0001772 (immune synapse), and GO:0042629 (mast cell granule) (Figure 6 and Supplementary Table 2).

In the GO and KEGG analyses of IRFs, the results showed multiple genes that are closely related to interferons, such as GO:0060333 (interference gamma mediated signaling path), GO:0060337 (type I interference signaling path), GO:0071357 (cellular response to type I interference), and GO:0034340 (response to type I interference) (Figure 6 and Supplementary Table 3).

**Figure 7:**
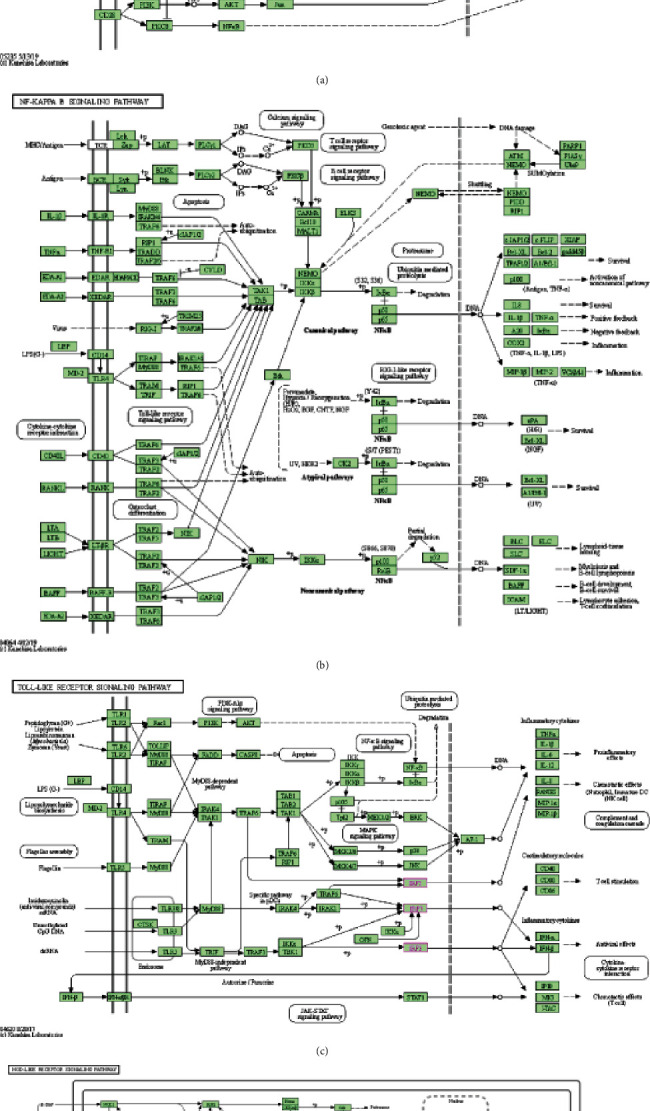
Pathways related to the expression of genes which closely associated with IRFs and pathways related to the expression of IRFs. (a) PD-L1 expression and PD-1 checkpoint pathway. (b) NF-kappa B signaling pathway. (c) Toll-like receptor signaling pathway. (d) NOD-like receptor signaling pathway.

KEGG analysis defined pathways associated with changes in IRFs and with neighboring genes that frequently changed adjacent gene functions. KEGG analysis evaluated the gene pathway that is associated with genes having expression closely correlated with IRF expression and identified hsa05235 (PD-L1 expression and PD-1 checkpoint pathway in cancer) and hsa04064 (NF-kappa B signaling pathway) pathways. Furthermore, KEGG analysis evaluated the pathways related to the expression of IRFs and identified hsa04620 (Toll-like receptor signaling pathway) and hsa04621 (NOD-like receptor signaling pathway). These pathways are all involved in the occurrence and pathogenesis of EC (Figure 7).

### 3.4. Correlation between IRF Expression and Immune Cell Infiltration

**Figure 8:**
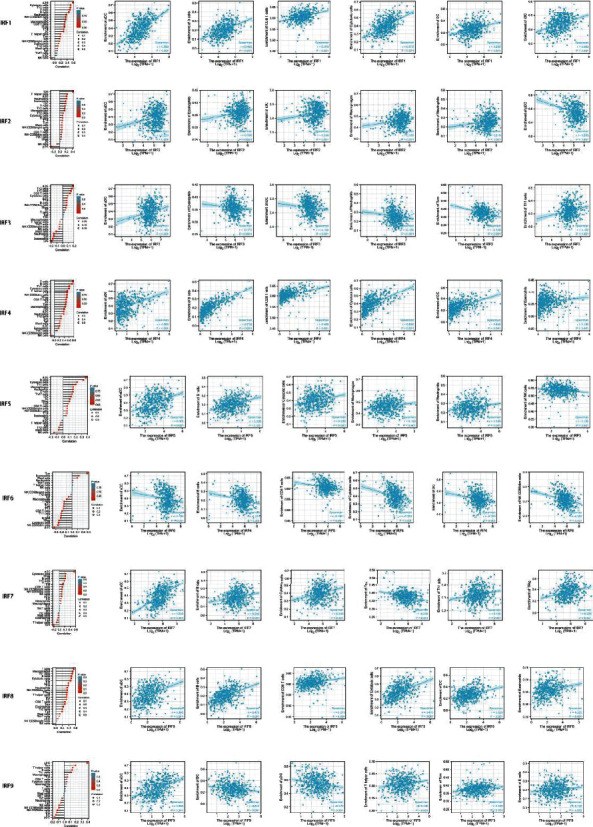
Correlation between immune cell infiltration and mRNA expression of IRFs and analysis of infiltration of the top six immune cells for each IRFs.

According to the GO and GSEA results, we hypothesized that IRFs may be involved in tumor immune responses. Therefore, we used ssGSEA to analyze the correlation between IRF mRNA expression and immune cell infiltration. The correlation between immune cell infiltration and IRF mRNA expression is shown in Figure 8. Subsequently, we analyzed the effect of the mRNA level of each IRF on the infiltration of the top six immune cells (Figure 8).

Detailed data on the relationship between IRF and immune cell infiltration are presented in Supplementary Table 4.

These data indicated that IRF may play a specific role in immune infiltration in EC.

## 4. Discussion

IRFs show an imbalance expression and played different roles in the occurrence and development of in many different cancers [24–39]. IRF6 might enhance chemotherapeutic sensitivity of cisplatin in colorectal cancer [36]. IRF4 was overexpressed and activated the cancer progression by Notch signaling pathway in human non-small-cell lung cancer [37]. IRF4 might be a novel regulator of PMN-MDSC development in cancer [38]. The miR-320/IRF6 signaling axis played an important role in pulmonary canceration [39]. Although a role for IRFs in the tumorigenesis and prognosis of several cancers has been partially elucidated, no further bioinformatic analysis of EC has been conducted. Moreover, the role of IRFs in EC has not been studied in recent years. The current study is the first to investigate the mRNA expression of different IRFs and correlate expression levels with prognostic value (OS, DSS, and PFI) and immune cell infiltration in EC. We believe that our findings will contribute to the available knowledge, improve treatment design, and improve the accuracy of prognosis in patients with EC.

In a previous study on IRFs and EC, only IRF1 and EC were studied, elucidating that the expression of IRF1 is downregulated in human endometrioid adenocarcinoma compared with a normal endometrium and a postmenopausal endometrium [16]. Reports of downregulation of IRF1 in endometrial tumorigenesis are common; however, derepression of IRF1 may occur in a subset of tumors, an event which is associated with thymidine phosphorylase upregulation and aggressive tumor behavior [15]. As per our analysis, no significant differences were identified in the expression of IRF1 in both paired and unpaired samples when assessing cancer stage and prognosis. We concluded that IRF1 expression is not closely correlated to the pathological stage and prognosis of EC.

IRF2–9 have not been studied in EC. According to our findings, IRF3, IRF5, IRF6, IRF7, and IRF9 were significantly overexpressed in EC, and IRF2 and IRF8 were significantly underexpressed in EC. However, there was no difference in the expression of IRF5 or IRF9 in EC paired samples. We found that only IRF3 levels were correlated with the clinical stage of EC.

Among the highly expressed IRFs identified in EC, only IRF5 was associated with OS, and among IRFs with low level of expression in EC, only IRF2 was associated with DSS. There was no difference in IRF4 expression in paired or unpaired samples. However, IRF4 is closely correlated to OS, DSS, and PFI in patients with EC. These results also demonstrate the value of IRF2, IRF4, and IRF5 as prognostic indicators. However, our data indicates that IRF2, IRF6, IRF7, and IRF8 are more valuable as diagnostic indicators. According to the results of immune infiltration analysis, there is a close correlation between IRFs and immune cell infiltration in EC. Similarly, according to the results of GO and KEGG analyses, IRFs and closely associated coexpressed genes are involved in PD-L1 expression and the PD-1 checkpoint pathway. This also indicates the putative value of PD-L1 and PD-1 inhibitors in the treatment of EC.

## 5. Conclusion

In this study, we systematically analyzed the expression, prognostic value, and immune cell infiltration of IRFs in EC. We also had a thorough understanding of the heterogeneity and complexity of endometrial carcinoma molecular biology. Our results indicate that the altered expression levels of IRF2, IRF3, IRF5, IRF6, IRF7, IRF8, and IRF9 may play important roles in the occurrence and development of EC. IRF2, IRF6, IRF7, and IRF8 may be used as molecular markers to identify patients with EC, and IRF2, IRF4, and IRF5 may be used as prognostic indicators. However, the specific mechanism underlying the role of IRFs in EC needs to be further studied.

## Data Availability

The data sets used and/or analyzed in this study are from public databases and can be obtained from the corresponding authors upon reasonable request (https://www.cancer.gov/aboutnci/organization/ccg/research/structural-genomics/tcga).

## Abbreviations

DAVID: Database for Annotation, Visualization, and Integrated Discovery
DBD: DNA-binding domain
DEG: Differentially expressed gene
DSS: Disease-specific survival
GEPIA: Gene Expression Profiling Interactive Analysis
GO: Gene Ontology
GSEA: Gene set enrichment analysis
HPA: Human Protein Atlas
IAD: IRF-association domain
IRF: Interferon regulatory factor
ISG: Interferon-stimulated gene
ISRE: Interferon-stimulated response element
KEGG: Kyoto Encyclopedia of Genes and Genomes
OS: Overall survival
PFI: Progression-free interval
ROC: Receiver operating characteristic
TCGA: The Cancer Genome Atlas.

## Additional Points

*Research Highlights*. IRFs may be used as biomarkers and prognostic indicators in endometrial carcinoma.

## Supplementary Materials

Supplementary 1 Supplementary Table 1: the 152 most frequently altered neighboring genes of IRFs.

Supplementary 2 Supplementary Table 2: Gene Ontology (GO) and Kyoto Encyclopedia of Genes and Genomes (KEGG) analyses of functions of genes that are significantly associated with IRFs alterations.

Supplementary 3 Supplementary Table 3: Gene Ontology (GO) and Kyoto Encyclopedia of Genes and Genomes (KEGG) analyses of functions of IRFs genes.

Supplementary 4 Supplementary Table 4: the relationship between IRFs and immune cell infiltration.
