# Supplementary material for: A Comprehensive Analysis of Interferon Regulatory Factor Expression: Correlation with Immune Cell Infiltration and Patient Prognosis in Endometrial Carcinoma
Source: Biomed Res Int. 2022 Aug 8;2022:7948898. doi: 10.1155/2022/7948898 (PMC9381850; doi:10.1155/2022/7948898)
Supplement: Supplementary 1 — Supplementary Table 1: the 152 most frequently altered neighboring genes of IRFs. [file 7948898.f1.pdf]

id
PSMB9
IRF1-AS1
APOL3
IL15RA
ARHGAP9
CD7
PTPN7
BATF2
IL2RG
SASH3
CD48
WAS
PSMB10
APOBEC3G
CD247
LAPTM5
MYO1F
NKG7
LAT2
SLC15A3
SP140
CARD16
LSP1
KLRK1
CD3E
CCL5
FERMT3
NCF1C
HLA-F
DOK2
CD3D
RASAL3
SLA2
NCF1
CORO1A
CCL4
CYTH4
FCER1G
APOL2
NCF4
UBASH3A
BTK
SIRPG
LST1
RAC2
ACAP1
IL18RAP
LAIR1
ABI3
HCST
SLAMF7
CCL4L1
TNFAIP8L2
TESPA1
GZMA
APOBEC3D
TYROBP

PSTPIP1
TARP
GPR65
GBP2
TRAF3IP3
CD6
C1QA
CST7
AIM2
CD244
LTA
AIF1
GNGT2
GPSM3
SCML4
LTB
TMC8
PIK3CD
CD52
APOBR
ZBP1
XAF1
FUT7
C1QB
CD300LF
CTSW
TNFRSF14
CXCR3
LCK
GZMM
TBX21
ARHGDIB
GIMAP5
CD70
PIK3R6
ZNF683
TMIGD2
CLECL1
ARHGAP15
C1ORF162
FCGR1A
MEI1
LILRB2
PTPRCAP
FCGR1CP
GIMAP1
FAM78A
ADAP2
ZAP70
GAB3
FMNL1
LY86
XCL2
PLCB2
MILR1
CXCR5
GIMAP4
ARHGAP25

SP140L
OSCAR
BATF
PRAM1
GNLY
TNFRSF13B
TNFSF13B
TNFRSF14-AS1
IL18BP
SELL
TNFRSF17
CLEC2B
MATK
SAMD3
DPEP2
ARL11
ITGB7
LILRA5
ISG20
CLEC4E
DOK3
DDX60
APOBEC3H
HTRA4
ARHGAP45
C11ORF21
MLKL
TCIRG1
HSH2D
RHOG
FLT3
PTCRA
MS4A14
LIPC
RENBP
LIME1
CPVL
