# Supplementary material for: A Comprehensive Analysis of Interferon Regulatory Factor Expression: Correlation with Immune Cell Infiltration and Patient Prognosis in Endometrial Carcinoma
Source: Biomed Res Int. 2022 Aug 8;2022:7948898. doi: 10.1155/2022/7948898 (PMC9381850; doi:10.1155/2022/7948898)
Supplement: Supplementary 2 — Supplementary Table 2: Gene Ontology (GO) and Kyoto Encyclopedia of Genes and Genomes (KEGG) analyses of functions of genes that are significantly associated with IRFs alterations. [file 7948898.f2.pdf]

ONTOLOGY	ID	Description	GeneRatio	BgRatio	pvalue	p.adjust	qvalue
BP	GO:0042110	T cell activati	34/137	464/18670	1.3676E-24	2.2689E-21	1.6296E-21
BP	GO:0051249	regulation of	29/137	485/18670	1.4773E-18	1.2254E-15	8.8015E-16
BP	GO:0007159	leukocyte cel	25/137	337/18670	2.7964E-18	1.5464E-15	1.1107E-15
BP	GO:0050863	regulation of	23/137	314/18670	9.4507E-17	3.7777E-14	2.7133E-14
BP	GO:0050867	positive regu	25/137	394/18670	1.1386E-16	3.7777E-14	2.7133E-14
BP	GO:0051251	positive regu	23/137	334/18670	3.6523E-16	9.3927E-14	6.7464E-14
BP	GO:0070661	leukocyte pro	22/137	298/18670	3.9632E-16	9.3927E-14	6.7464E-14
BP	GO:0050870	positive regu	19/137	202/18670	5.4721E-16	1.0167E-13	7.3027E-14
BP	GO:0002696	positive regu	24/137	380/18670	5.5157E-16	1.0167E-13	7.3027E-14
BP	GO:0046651	lymphocyte p	21/137	272/18670	7.9726E-16	1.3056E-13	9.3778E-14
BP	GO:0032943	mononuclear	21/137	274/18670	9.2421E-16	1.3056E-13	9.3778E-14
BP	GO:0050670	regulation of	19/137	208/18670	9.4441E-16	1.3056E-13	9.3778E-14
BP	GO:0032944	regulation of	19/137	209/18670	1.0326E-15	1.3177E-13	9.4645E-14
BP	GO:1903039	positive regu	19/137	218/18670	2.2577E-15	2.6754E-13	1.9216E-13
BP	GO:0070663	regulation of	19/137	222/18670	3.1598E-15	3.4947E-13	2.5101E-13
BP	GO:1903037	regulation of	21/137	304/18670	7.4039E-15	7.6769E-13	5.514E-13
BP	GO:0022409	positive regu	19/137	255/18670	3.9979E-14	3.9015E-12	2.8022E-12
BP	GO:0022407	regulation of	22/137	403/18670	1.9965E-13	1.8299E-11	1.3143E-11
BP	GO:0042102	positive regu	13/137	95/18670	2.0957E-13	1.8299E-11	1.3143E-11
BP	GO:0050900	leukocyte mi	24/137	499/18670	2.2516E-13	1.8677E-11	1.3415E-11
BP	GO:0042098	T cell prolifer	16/137	184/18670	4.2999E-13	3.3969E-11	2.4398E-11
BP	GO:0042129	regulation of	15/137	156/18670	5.5222E-13	4.1643E-11	2.991E-11
BP	GO:0002449	lymphocyte m	19/137	352/18670	1.2279E-11	8.7407E-10	6.2781E-10
BP	GO:0050671	positive regu	13/137	130/18670	1.2645E-11	8.7407E-10	6.2781E-10
BP	GO:0032946	positive regu	13/137	131/18670	1.3951E-11	9.258E-10	6.6495E-10
BP	GO:0030217	T cell differen	16/137	240/18670	2.5094E-11	1.6012E-09	1.1501E-09
BP	GO:0070665	positive regu	13/137	139/18670	2.9757E-11	1.8284E-09	1.3133E-09
BP	GO:0072676	lymphocyte m	12/137	111/18670	3.178E-11	1.8829E-09	1.3524E-09
BP	GO:0030098	lymphocyte d	18/137	353/18670	1.1275E-10	6.4501E-09	4.6328E-09
BP	GO:0045785	positive regu	19/137	403/18670	1.2534E-10	6.9313E-09	4.9784E-09
BP	GO:0002697	regulation of	20/137	458/18670	1.5212E-10	8.1409E-09	5.8472E-09
BP	GO:0001819	positive regu	20/137	464/18670	1.9133E-10	9.919E-09	7.1244E-09
BP	GO:0002429	immune resp	20/137	473/18670	2.6811E-10	1.3479E-08	9.6811E-09
BP	GO:0050852	T cell recepto	14/137	202/18670	2.8257E-10	1.3788E-08	9.903E-09
BP	GO:0071356	cellular respo	16/137	291/18670	4.3807E-10	2.0764E-08	1.4914E-08
BP	GO:0002695	negative regu	13/137	175/18670	5.3689E-10	2.4742E-08	1.7771E-08
BP	GO:0034612	response to t	16/137	312/18670	1.2076E-09	5.4147E-08	3.8891E-08
BP	GO:0002460	adaptive imm	17/137	361/18670	1.2988E-09	5.6703E-08	4.0727E-08
BP	GO:0050851	antigen recep	16/137	316/18670	1.4518E-09	6.1758E-08	4.4358E-08
BP	GO:0050866	negative regu	13/137	199/18670	2.6015E-09	1.079E-07	7.7498E-08
BP	GO:0042113	B cell activati	15/137	310/18670	9.0066E-09	3.6444E-07	2.6176E-07
BP	GO:0030595	leukocyte che	13/137	224/18670	1.0857E-08	4.2887E-07	3.0804E-07
BP	GO:0072678	T cell migrati	8/137	65/18670	2.4292E-08	9.3721E-07	6.7316E-07
BP	GO:0002703	regulation of	12/137	201/18670	2.9925E-08	1.1283E-06	8.1042E-07
BP	GO:0033209	tumor necros	11/137	167/18670	4.2212E-08	1.5562E-06	1.1178E-06
BP	GO:0060326	cell chemota	14/137	304/18670	5.3691E-08	1.9364E-06	1.3908E-06
BP	GO:0002699	positive regu	12/137	216/18670	6.6211E-08	2.3371E-06	1.6786E-06
BP	GO:0046631	alpha-beta T	10/137	138/18670	7.2424E-08	2.5032E-06	1.7979E-06
BP	GO:0051250	negative regu	10/137	146/18670	1.2337E-07	4.1769E-06	3E-06

BP	GO:0002706	regulation of	10/137	149/18670	1.4937E-07	4.9562E-06	3.5598E-06
BP	GO:0001776	leukocyte ho	8/137	86/18670	2.2443E-07	7.1601E-06	5.1427E-06
BP	GO:0030101	natural killer	8/137	86/18670	2.2443E-07	7.1601E-06	5.1427E-06
BP	GO:0045730	respiratory b	6/137	37/18670	2.6968E-07	8.4259E-06	6.0519E-06
BP	GO:0045576	mast cell acti	7/137	60/18670	2.7426E-07	8.4259E-06	6.0519E-06
BP	GO:0002819	regulation of	10/137	160/18670	2.9067E-07	8.7675E-06	6.2973E-06
BP	GO:0002683	negative regu	16/137	463/18670	2.9874E-07	8.8502E-06	6.3566E-06
BP	GO:0048247	lymphocyte c	7/137	64/18670	4.3052E-07	1.253E-05	9E-06
BP	GO:0032103	positive regu	13/137	323/18670	7.6356E-07	2.1841E-05	1.5687E-05
BP	GO:0035747	natural killer	4/137	11/18670	8.7984E-07	2.474E-05	1.777E-05
BP	GO:0002708	positive regu	8/137	105/18670	1.0493E-06	2.9014E-05	2.084E-05
BP	GO:0043303	mast cell deg	6/137	47/18670	1.1728E-06	3.1898E-05	2.291E-05
BP	GO:0001909	leukocyte me	8/137	107/18670	1.2117E-06	3.2424E-05	2.3288E-05
BP	GO:0002279	mast cell acti	6/137	48/18670	1.3324E-06	3.4843E-05	2.5026E-05
BP	GO:0002440	production o	12/137	286/18670	1.3442E-06	3.4843E-05	2.5026E-05
BP	GO:0002448	mast cell me	6/137	49/18670	1.5092E-06	3.8519E-05	2.7666E-05
BP	GO:0043547	positive regu	14/137	405/18670	1.7175E-06	4.3171E-05	3.1007E-05
BP	GO:0042590	antigen proc	7/137	79/18670	1.8335E-06	4.5399E-05	3.2608E-05
BP	GO:0006968	cellular defer	6/137	53/18670	2.4188E-06	5.8646E-05	4.2122E-05
BP	GO:0043087	regulation of	15/137	479/18670	2.4392E-06	5.8646E-05	4.2122E-05
BP	GO:0006909	phagocytosis	13/137	369/18670	3.34E-06	7.8503E-05	5.6385E-05
BP	GO:0031295	T cell costimu	6/137	56/18670	3.3597E-06	7.8503E-05	5.6385E-05
BP	GO:0031294	lymphocyte c	6/137	57/18670	3.7324E-06	8.6002E-05	6.1771E-05
BP	GO:0001906	cell killing	9/137	168/18670	4.1765E-06	9.4916E-05	6.8173E-05
BP	GO:0043368	positive T cel	5/137	34/18670	4.6365E-06	0.00010395	7.4659E-05
BP	GO:0045089	positive regu	13/137	381/18670	4.7312E-06	0.00010465	7.5168E-05
BP	GO:0042267	natural killer	6/137	60/18670	5.0574E-06	0.0001104	7.9293E-05
BP	GO:0019724	B cell mediat	10/137	221/18670	5.4556E-06	0.00011704	8.4062E-05
BP	GO:0046634	regulation of	7/137	93/18670	5.5026E-06	0.00011704	8.4062E-05
BP	GO:0045088	regulation of	14/137	452/18670	6.1083E-06	0.00012828	9.2134E-05
BP	GO:0002705	positive regu	8/137	133/18670	6.2091E-06	0.00012876	9.2483E-05
BP	GO:2000401	regulation of	6/137	63/18670	6.7412E-06	0.00013753	9.8783E-05
BP	GO:0002474	antigen proc	7/137	96/18670	6.7979E-06	0.00013753	9.8783E-05
BP	GO:2000403	positive regu	5/137	37/18670	7.1361E-06	0.00014264	0.00010245
BP	GO:0071346	cellular respo	9/137	180/18670	7.3238E-06	0.00014431	0.00010365
BP	GO:0002228	natural killer	6/137	64/18670	7.394E-06	0.00014431	0.00010365
BP	GO:0002824	positive regu	7/137	100/18670	8.9101E-06	0.00017188	0.00012346
BP	GO:0002367	cytokine proc	7/137	102/18670	1.0155E-05	0.00019364	0.00013908
BP	GO:0030593	neutrophil ch	7/137	104/18670	1.154E-05	0.00021756	0.00015626
BP	GO:0002822	regulation of	8/137	145/18670	1.1723E-05	0.00021852	0.00015695
BP	GO:0002821	positive regu	7/137	105/18670	1.229E-05	0.00022654	0.00016271
BP	GO:0002456	T cell mediat	7/137	106/18670	1.3079E-05	0.00023844	0.00017126
BP	GO:0002685	regulation of	9/137	196/18670	1.4522E-05	0.00026187	0.00018809
BP	GO:0032945	negative regu	6/137	73/18670	1.5905E-05	0.00028071	0.00020162
BP	GO:0050672	negative regu	6/137	73/18670	1.5905E-05	0.00028071	0.00020162
BP	GO:0034341	response to i	9/137	199/18670	1.6391E-05	0.00028624	0.00020559
BP	GO:0032418	lysosome loc	6/137	74/18670	1.7205E-05	0.00029733	0.00021355
BP	GO:0042119	neutrophil ac	14/137	498/18670	1.8163E-05	0.00031064	0.00022311
BP	GO:0002479	antigen proc	6/137	75/18670	1.8589E-05	0.00031469	0.00022603
BP	GO:0032609	interferon-ga	7/137	113/18670	1.9863E-05	0.00033286	0.00023908

BP	GO:0002688	regulation of	7/137	114/18670	2.1035E-05	0.00034896	0.00025064
BP	GO:0070664	negative regu	6/137	78/18670	2.3289E-05	0.00038255	0.00027476
BP	GO:0045058	T cell selectio	5/137	47/18670	2.3677E-05	0.00038511	0.0002766
BP	GO:1990266	neutrophil m	7/137	118/18670	2.6305E-05	0.00042369	0.00030431
BP	GO:0071621	granulocyte c	7/137	123/18670	3.4377E-05	0.00054838	0.00039387
BP	GO:0045619	regulation of	8/137	169/18670	3.5449E-05	0.00055646	0.00039968
BP	GO:0002718	regulation of	6/137	84/18670	3.5555E-05	0.00055646	0.00039968
BP	GO:0043901	negative regu	8/137	171/18670	3.855E-05	0.0005977	0.0004293
BP	GO:1901623	regulation of	4/137	27/18670	4.2713E-05	0.00065612	0.00047126
BP	GO:0002690	positive regu	6/137	87/18670	4.3383E-05	0.0006603	0.00047426
BP	GO:0002687	positive regu	7/137	128/18670	4.4383E-05	0.00066647	0.00047869
BP	GO:0019882	antigen proc	9/137	226/18670	4.4592E-05	0.00066647	0.00047869
BP	GO:0070098	chemokine-m	6/137	88/18670	4.6279E-05	0.00068551	0.00049237
BP	GO:0010818	T cell chemot	4/137	28/18670	4.9549E-05	0.00072745	0.00052249
BP	GO:0002285	lymphocyte a	8/137	181/18670	5.7667E-05	0.0008319	0.00059752
BP	GO:0048015	phosphatidyl	8/137	181/18670	5.7667E-05	0.0008319	0.00059752
BP	GO:0033632	regulation of	3/137	11/18670	6.1091E-05	0.00087371	0.00062754
BP	GO:0048017	inositol lipid-	8/137	184/18670	6.4742E-05	0.00091411	0.00065656
BP	GO:0046640	regulation of	4/137	30/18670	6.5569E-05	0.00091411	0.00065656
BP	GO:0050869	negative regu	4/137	30/18670	6.5569E-05	0.00091411	0.00065656
BP	GO:0045621	positive regu	6/137	94/18670	6.7077E-05	0.00092734	0.00066606
BP	GO:0050663	cytokine secr	9/137	240/18670	7.0935E-05	0.00095995	0.00068949
BP	GO:0002702	positive regu	6/137	95/18670	7.1172E-05	0.00095995	0.00068949
BP	GO:0042100	B cell prolifer	6/137	95/18670	7.1172E-05	0.00095995	0.00068949
BP	GO:0045071	negative regu	5/137	59/18670	7.2017E-05	0.00096352	0.00069205
BP	GO:0038093	Fc receptor s	9/137	241/18670	7.3234E-05	0.00097123	0.00069759
BP	GO:2000406	positive regu	4/137	31/18670	7.4856E-05	0.00097123	0.00069759
BP	GO:0002700	regulation of	7/137	139/18670	7.4935E-05	0.00097123	0.00069759
BP	GO:0045580	regulation of	7/137	139/18670	7.4935E-05	0.00097123	0.00069759
BP	GO:0018108	peptidyl-tyro	11/137	363/18670	7.6865E-05	0.0009883	0.00070984
BP	GO:1990868	response to c	6/137	97/18670	7.9959E-05	0.0009883	0.00070984
BP	GO:1990869	cellular respo	6/137	97/18670	7.9959E-05	0.0009883	0.00070984
BP	GO:0002863	positive regu	3/137	12/18670	8.1018E-05	0.0009883	0.00070984
BP	GO:0006216	cytidine catal	3/137	12/18670	8.1018E-05	0.0009883	0.00070984
BP	GO:0009972	cytidine dear	3/137	12/18670	8.1018E-05	0.0009883	0.00070984
BP	GO:0016554	cytidine to ur	3/137	12/18670	8.1018E-05	0.0009883	0.00070984
BP	GO:0046087	cytidine meta	3/137	12/18670	8.1018E-05	0.0009883	0.00070984
BP	GO:0097530	granulocyte r	7/137	141/18670	8.1999E-05	0.00099296	0.0007132
BP	GO:0018212	peptidyl-tyro	11/137	366/18670	8.2702E-05	0.00099422	0.0007141
BP	GO:0030888	regulation of	5/137	61/18670	8.458E-05	0.00100802	0.00072401
BP	GO:0002323	natural killer	4/137	32/18670	8.5065E-05	0.00100802	0.00072401
BP	GO:0048872	homeostasis	9/137	246/18670	8.5695E-05	0.00100828	0.0007242
BP	GO:0048525	negative regu	6/137	99/18670	8.959E-05	0.00104668	0.00075178
BP	GO:0002260	lymphocyte h	5/137	62/18670	9.1461E-05	0.00105371	0.00075683
BP	GO:0032623	interleukin-2	5/137	62/18670	9.1461E-05	0.00105371	0.00075683
BP	GO:1902107	positive regu	7/137	144/18670	9.3605E-05	0.00107097	0.00076923
BP	GO:0032633	interleukin-4	4/137	33/18670	9.625E-05	0.00108624	0.0007802
BP	GO:0046633	alpha-beta T	4/137	33/18670	9.625E-05	0.00108624	0.0007802
BP	GO:0046632	alpha-beta T	6/137	101/18670	0.00010012	0.00112231	0.0008061
BP	GO:0045006	DNA deamina	3/137	13/18670	0.00010476	0.00115862	0.00083218

BP	GO:0045059	positive thym	3/137	13/18670	0.00010476	0.00115862	0.00083218
BP	GO:0014065	phosphatidyl	7/137	148/18670	0.00011113	0.00122094	0.00087694
BP	GO:0002548	monocyte ch	5/137	65/18670	0.00011471	0.0012438	0.00089336
BP	GO:0032729	positive regu	5/137	65/18670	0.00011471	0.0012438	0.00089336
BP	GO:0045869	negative regu	3/137	14/18670	0.00013261	0.00141224	0.00101434
BP	GO:0046133	pyrimidine ri	3/137	14/18670	0.00013261	0.00141224	0.00101434
BP	GO:0002573	myeloid leuk	8/137	204/18670	0.0001328	0.00141224	0.00101434
BP	GO:0002714	positive regu	4/137	37/18670	0.00015186	0.00159449	0.00114525
BP	GO:0002891	positive regu	4/137	37/18670	0.00015186	0.00159449	0.00114525
BP	GO:0097529	myeloid leuk	8/137	210/18670	0.00016207	0.00169105	0.0012146
BP	GO:0050766	positive regu	5/137	70/18670	0.00016322	0.00169243	0.00121559
BP	GO:1902105	regulation of	9/137	272/18670	0.00018296	0.00188531	0.00135413
BP	GO:0051056	regulation of	10/137	338/18670	0.00019908	0.00202937	0.0014576
BP	GO:0043900	regulation of	11/137	405/18670	0.00020072	0.00202937	0.0014576
BP	GO:0030889	negative regu	3/137	16/18670	0.00020184	0.00202937	0.0014576
BP	GO:0033631	cell-cell adhe	3/137	16/18670	0.00020184	0.00202937	0.0014576
BP	GO:0002369	T cell cytokin	4/137	40/18670	0.00020658	0.00206459	0.00148289
BP	GO:0016064	immunoglob	8/137	218/18670	0.00020919	0.00206576	0.00148373
BP	GO:0019722	calcium-med	8/137	218/18670	0.00020919	0.00206576	0.00148373
BP	GO:0043312	neutrophil de	12/137	485/18670	0.00024312	0.00236502	0.00169868
BP	GO:0045091	regulation of	3/137	17/18670	0.00024377	0.00236502	0.00169868
BP	GO:0048535	lymph node (	3/137	17/18670	0.00024377	0.00236502	0.00169868
BP	GO:0033003	regulation of	4/137	42/18670	0.00025016	0.00239891	0.00172302
BP	GO:0097028	dendritic cell	4/137	42/18670	0.00025016	0.00239891	0.00172302
BP	GO:0002283	neutrophil ac	12/137	488/18670	0.00025718	0.00245211	0.00176123
BP	GO:0001910	regulation of	5/137	78/18670	0.00027168	0.00257549	0.00184985
BP	GO:0039692	single strand	3/137	18/18670	0.00029096	0.00274262	0.00196989
BP	GO:0042269	regulation of	4/137	44/18670	0.00029997	0.00279582	0.0020081
BP	GO:2000404	regulation of	4/137	44/18670	0.00029997	0.00279582	0.0020081
BP	GO:0014066	regulation of	6/137	124/18670	0.00030746	0.00284963	0.00204675
BP	GO:0002478	antigen proc	7/137	175/18670	0.00031189	0.00287462	0.0020647
BP	GO:0002446	neutrophil m	12/137	499/18670	0.00031481	0.0028855	0.00207251
BP	GO:0046641	positive regu	3/137	19/18670	0.00034366	0.0031155	0.00223771
BP	GO:2000010	positive regu	3/137	19/18670	0.00034366	0.0031155	0.00223771
BP	GO:0002437	inflammatory	4/137	46/18670	0.00035653	0.00317248	0.00227864
BP	GO:0002715	regulation of	4/137	46/18670	0.00035653	0.00317248	0.00227864
BP	GO:0043300	regulation of	4/137	46/18670	0.00035653	0.00317248	0.00227864
BP	GO:0071347	cellular respo	7/137	179/18670	0.0003576	0.00317248	0.00227864
BP	GO:0045582	positive regu	5/137	83/18670	0.00036287	0.00318516	0.00228775
BP	GO:2000106	regulation of	5/137	83/18670	0.00036287	0.00318516	0.00228775
BP	GO:0019884	antigen proc	7/137	182/18670	0.00039528	0.0034514	0.00247898
BP	GO:1903901	negative regu	5/137	85/18670	0.00040518	0.00351934	0.00252777
BP	GO:0001774	microglial cel	4/137	48/18670	0.00042033	0.00360976	0.00259271
BP	GO:0002269	leukocyte act	4/137	48/18670	0.00042033	0.00360976	0.00259271
BP	GO:0050864	regulation of	7/137	184/18670	0.00042212	0.00360976	0.00259271
BP	GO:1903708	positive regu	7/137	185/18670	0.00043607	0.00370997	0.00266469
BP	GO:0014068	positive regu	5/137	87/18670	0.00045112	0.00381837	0.00274255
BP	GO:0002438	acute inflam	3/137	21/18670	0.00046666	0.00385165	0.00276645
BP	GO:0016553	base convers	3/137	21/18670	0.00046666	0.00385165	0.00276645
BP	GO:0045061	thymic T cell	3/137	21/18670	0.00046666	0.00385165	0.00276645

BP	GO:0048245	eosinophil ch	3/137	21/18670	0.00046666	0.00385165	0.00276645
BP	GO:0140131	positive regu	3/137	21/18670	0.00046666	0.00385165	0.00276645
BP	GO:0050921	positive regu	6/137	135/18670	0.00048436	0.00397797	0.00285718
BP	GO:0048002	antigen proce	7/137	189/18670	0.00049563	0.00405047	0.00290925
BP	GO:0071674	mononuclear	5/137	90/18670	0.00052724	0.00427423	0.00306997
BP	GO:0002712	regulation of	4/137	51/18670	0.00053074	0.00427423	0.00306997
BP	GO:0002889	regulation of	4/137	51/18670	0.00053074	0.00427423	0.00306997
BP	GO:0050731	positive regu	7/137	192/18670	0.00054441	0.00436318	0.00313386
BP	GO:0002377	immunoglob	7/137	193/18670	0.0005615	0.00447848	0.00321667
BP	GO:0050715	positive regu	6/137	139/18670	0.00056536	0.00448768	0.00322328
BP	GO:0032653	regulation of	4/137	52/18670	0.00057172	0.00451658	0.00324404
BP	GO:0035710	CD4-positive,	5/137	92/18670	0.00058311	0.00458471	0.00329297
BP	GO:0002218	activation of	9/137	319/18670	0.00058684	0.00459227	0.00329841
BP	GO:0010528	regulation of	3/137	23/18670	0.00061476	0.00472166	0.00339134
BP	GO:0010529	negative regu	3/137	23/18670	0.00061476	0.00472166	0.00339134
BP	GO:0032753	positive regu	3/137	23/18670	0.00061476	0.00472166	0.00339134
BP	GO:0042454	ribonucleosid	3/137	23/18670	0.00061476	0.00472166	0.00339134
BP	GO:0002720	positive regu	4/137	54/18670	0.00066034	0.00504843	0.00362604
BP	GO:0045069	regulation of	5/137	95/18670	0.00067508	0.00509074	0.00365643
BP	GO:0060337	type I interfe	5/137	95/18670	0.00067508	0.00509074	0.00365643
BP	GO:0071357	cellular respo	5/137	95/18670	0.00067508	0.00509074	0.00365643
BP	GO:0009615	response to v	9/137	326/18670	0.00068511	0.005143	0.00369397
BP	GO:0002861	regulation of	3/137	24/18670	0.00069882	0.00515248	0.00370077
BP	GO:0042832	defense resp	3/137	24/18670	0.00069882	0.00515248	0.00370077
BP	GO:0046135	pyrimidine nu	3/137	24/18670	0.00069882	0.00515248	0.00370077
BP	GO:0032755	positive regu	5/137	96/18670	0.00070803	0.00515248	0.00370077
BP	GO:0002886	regulation of	4/137	55/18670	0.00070812	0.00515248	0.00370077
BP	GO:0032613	interleukin-1	4/137	55/18670	0.00070812	0.00515248	0.00370077
BP	GO:0061098	positive regu	4/137	55/18670	0.00070812	0.00515248	0.00370077
BP	GO:0001912	positive regu	4/137	56/18670	0.00075828	0.00549342	0.00394566
BP	GO:1903706	regulation of	11/137	475/18670	0.00076684	0.00553126	0.00397284
BP	GO:0031341	regulation of	5/137	98/18670	0.00077751	0.00555989	0.0039934
BP	GO:0050764	regulation of	5/137	98/18670	0.00077751	0.00555989	0.0039934
BP	GO:0001562	response to p	3/137	25/18670	0.00078987	0.00559995	0.00402217
BP	GO:0072677	eosinophil m	3/137	25/18670	0.00078987	0.00559995	0.00402217
BP	GO:0034340	response to t	5/137	99/18670	0.0008141	0.00574723	0.00412796
BP	GO:0070555	response to i	7/137	207/18670	0.00084876	0.00596647	0.00428543
BP	GO:0031663	lipopolysacch	4/137	58/18670	0.00086607	0.00603702	0.0043361
BP	GO:0061900	glial cell activ	4/137	58/18670	0.00086607	0.00603702	0.0043361
BP	GO:0080111	DNA demeth	3/137	26/18670	0.00088812	0.0061599	0.00442435
BP	GO:0032649	regulation of	5/137	101/18670	0.00089112	0.0061599	0.00442435
BP	GO:0050727	regulation of	11/137	485/18670	0.00090866	0.00625505	0.0044927
BP	GO:0050707	regulation of	7/137	210/18670	0.00092322	0.00632901	0.00454582
BP	GO:0050729	positive regu	6/137	153/18670	0.00093446	0.00637973	0.00458225
BP	GO:0007229	integrin-med	5/137	103/18670	0.00097344	0.00661862	0.00475383
BP	GO:0032673	regulation of	3/137	27/18670	0.0009938	0.00672942	0.00483341
BP	GO:0071887	leukocyte ap	5/137	104/18670	0.00101666	0.00685626	0.00492452
BP	GO:0032196	transposition	3/137	28/18670	0.00110711	0.00743598	0.0053409
BP	GO:0046635	positive regu	4/137	62/18670	0.00111329	0.00744258	0.00534564
BP	GO:0050920	regulation of	7/137	217/18670	0.00111706	0.00744258	0.00534564

BP	GO:0046637	regulation of	4/137	63/18670	0.00118207	0.00784419	0.0056341
BP	GO:0032635	interleukin-6	6/137	161/18670	0.0012158	0.00803592	0.00577181
BP	GO:0033032	regulation of	3/137	29/18670	0.00122825	0.008086	0.00580778
BP	GO:0045954	positive regu	3/137	30/18670	0.00135744	0.00890116	0.00639327
BP	GO:0002244	hematopoiet	6/137	166/18670	0.00142198	0.00928763	0.00667085
BP	GO:0002223	stimulatory (	5/137	113/18670	0.00147246	0.00957967	0.00688061
BP	GO:0002724	regulation of	3/137	31/18670	0.00149486	0.0096874	0.00695799
BP	GO:0002292	T cell differer	4/137	68/18670	0.00157113	0.01006372	0.00722828
BP	GO:0031343	positive regu	4/137	68/18670	0.00157113	0.01006372	0.00722828
BP	GO:0033627	cell adhesion	4/137	68/18670	0.00157113	0.01006372	0.00722828
BP	GO:0002758	innate immu	8/137	298/18670	0.00162186	0.01031032	0.0074054
BP	GO:0032814	regulation of	3/137	32/18670	0.0016407	0.01031032	0.0074054
BP	GO:0035510	DNA dealkyla	3/137	32/18670	0.0016407	0.01031032	0.0074054
BP	GO:0039694	viral RNA ger	3/137	32/18670	0.0016407	0.01031032	0.0074054
BP	GO:0071353	cellular respo	3/137	32/18670	0.0016407	0.01031032	0.0074054
BP	GO:0002220	innate immu	5/137	116/18670	0.0016534	0.01035088	0.00743454
BP	GO:0007265	Ras protein s	10/137	448/18670	0.00174702	0.01082715	0.00777661
BP	GO:0002709	regulation of	4/137	70/18670	0.00174905	0.01082715	0.00777661
BP	GO:0033077	T cell differer	4/137	70/18670	0.00174905	0.01082715	0.00777661
BP	GO:0002717	positive regu	3/137	33/18670	0.00179515	0.01103018	0.00792244
BP	GO:0033028	myeloid cell a	3/137	33/18670	0.00179515	0.01103018	0.00792244
BP	GO:0071216	cellular respo	7/137	236/18670	0.00180701	0.0110621	0.00794537
BP	GO:0002532	production o	4/137	72/18670	0.00194049	0.01181434	0.00848566
BP	GO:0009164	nucleoside ca	3/137	34/18670	0.00195837	0.01181434	0.00848566
BP	GO:0016601	Rac protein s	3/137	34/18670	0.00195837	0.01181434	0.00848566
BP	GO:0070670	response to i	3/137	34/18670	0.00195837	0.01181434	0.00848566
BP	GO:0006801	superoxide m	4/137	73/18670	0.00204145	0.01227088	0.00881358
BP	GO:0006874	cellular calciu	10/137	458/18670	0.00205302	0.01229589	0.00883154
BP	GO:0019079	viral genome	5/137	122/18670	0.00206355	0.01231447	0.00884488
BP	GO:0046131	pyrimidine ri	3/137	35/18670	0.00213056	0.01266879	0.00909938
BP	GO:0043367	CD4-positive,	4/137	74/18670	0.00214598	0.01271491	0.0091325
BP	GO:0150076	neuroinflamr	4/137	75/18670	0.00225414	0.01330826	0.00955867
BP	GO:0032733	positive regu	3/137	36/18670	0.00231186	0.01360063	0.00976867
BP	GO:0007204	positive regu	8/137	319/18670	0.00247677	0.0145193	0.01042851
BP	GO:0055074	calcium ion h	10/137	471/18670	0.00251412	0.01468635	0.01054849
BP	GO:0050853	B cell recepto	5/137	129/18670	0.00263071	0.01531348	0.01099893
BP	GO:0002517	T cell toleran	2/137	11/18670	0.00281521	0.01616065	0.01160741
BP	GO:0045060	negative thyr	2/137	11/18670	0.00281521	0.01616065	0.01160741
BP	GO:0070673	response to i	2/137	11/18670	0.00281521	0.01616065	0.01160741
BP	GO:0098883	synapse prur	2/137	11/18670	0.00281521	0.01616065	0.01160741
BP	GO:0050730	regulation of	7/137	256/18670	0.00285145	0.01631227	0.01171631
BP	GO:0050856	regulation of	3/137	39/18670	0.0029121	0.01654514	0.01188357
BP	GO:0072529	pyrimidine-co	3/137	39/18670	0.0029121	0.01654514	0.01188357
BP	GO:2000008	regulation of	3/137	40/18670	0.00313147	0.01773075	0.01273513
BP	GO:0002551	mast cell che	2/137	12/18670	0.00336204	0.01853034	0.01330944
BP	GO:0043383	negative T ce	2/137	12/18670	0.00336204	0.01853034	0.01330944
BP	GO:0043922	negative regu	2/137	12/18670	0.00336204	0.01853034	0.01330944
BP	GO:0070486	leukocyte ag	2/137	12/18670	0.00336204	0.01853034	0.01330944
BP	GO:0072540	T-helper 17 c	2/137	12/18670	0.00336204	0.01853034	0.01330944
BP	GO:0097340	inhibition of	2/137	12/18670	0.00336204	0.01853034	0.01330944

BP	GO:0097341	zymogen inh	2/137	12/18670	0.00336204	0.01853034	0.01330944
BP	GO:0097531	mast cell mig	2/137	12/18670	0.00336204	0.01853034	0.01330944
BP	GO:0072503	cellular dival	10/137	493/18670	0.00348131	0.01912417	0.01373596
BP	GO:0002831	regulation of	5/137	139/18670	0.00362632	0.01985502	0.01426089
BP	GO:0032760	positive regu	4/137	86/18670	0.00370195	0.02020241	0.0145104
BP	GO:0050850	positive regu	3/137	43/18670	0.00384943	0.02086993	0.01498986
BP	GO:1901658	glycosyl com	3/137	43/18670	0.00384943	0.02086993	0.01498986
BP	GO:0002864	regulation of	2/137	13/18670	0.00395428	0.02116177	0.01519947
BP	GO:0033004	negative regu	2/137	13/18670	0.00395428	0.02116177	0.01519947
BP	GO:0035723	interleukin-1	2/137	13/18670	0.00395428	0.02116177	0.01519947
BP	GO:0071350	cellular respo	2/137	13/18670	0.00395428	0.02116177	0.01519947
BP	GO:0038094	Fc-gamma re	5/137	142/18670	0.00397149	0.02118556	0.01521656
BP	GO:0061097	regulation of	4/137	88/18670	0.00401971	0.02130577	0.0153029
BP	GO:1903557	positive regu	4/137	88/18670	0.00401971	0.02130577	0.0153029
BP	GO:0071222	cellular respo	6/137	205/18670	0.00407316	0.02152031	0.01545699
BP	GO:0032651	regulation of	4/137	90/18670	0.00435545	0.02291863	0.01646134
BP	GO:0050792	regulation of	6/137	208/18670	0.00437066	0.02291863	0.01646134
BP	GO:0002820	negative regu	3/137	45/18670	0.00437927	0.02291863	0.01646134
BP	GO:0050862	positive regu	2/137	14/18670	0.00459122	0.02387724	0.01714986
BP	GO:0070672	response to i	2/137	14/18670	0.00459122	0.02387724	0.01714986
BP	GO:0033628	regulation of	3/137	46/18670	0.00465993	0.02408354	0.01729803
BP	GO:0050706	regulation of	3/137	46/18670	0.00465993	0.02408354	0.01729803
BP	GO:0071219	cellular respo	6/137	212/18670	0.00479193	0.02468884	0.0177328
BP	GO:1903900	regulation of	5/137	149/18670	0.0048674	0.02500005	0.01795632
BP	GO:0051480	regulation of	8/137	357/18670	0.00488514	0.02501374	0.01796615
BP	GO:2000107	negative regu	3/137	47/18670	0.00495123	0.02527411	0.01815316
BP	GO:0008360	regulation of	5/137	150/18670	0.00500617	0.02547619	0.01829831
BP	GO:0072593	reactive oxyg	7/137	284/18670	0.00502539	0.02549577	0.01831237
BP	GO:0019932	second-mess	9/137	439/18670	0.00514036	0.02599955	0.01867421
BP	GO:0042116	macrophage	4/137	95/18670	0.00527639	0.02660484	0.01910896
BP	GO:0032675	regulation of	5/137	152/18670	0.0052921	0.02660484	0.01910896
BP	GO:0045807	positive regu	5/137	153/18670	0.00543932	0.02726232	0.0195812
BP	GO:0030316	osteoclast di	4/137	97/18670	0.00567858	0.02837581	0.02038096
BP	GO:0002526	acute inflamm	6/137	220/18670	0.00572317	0.02851271	0.02047929
BP	GO:0050848	regulation of	4/137	98/18670	0.00588714	0.02908239	0.02088847
BP	GO:0034656	nucleobase-c	3/137	50/18670	0.0058901	0.02908239	0.02088847
BP	GO:0046854	phosphatidyl	3/137	50/18670	0.0058901	0.02908239	0.02088847
BP	GO:0043903	regulation of	6/137	222/18670	0.0059753	0.02917376	0.0209541
BP	GO:0002295	T-helper cell	2/137	16/18670	0.00599654	0.02917376	0.0209541
BP	GO:0002643	regulation of	2/137	16/18670	0.00599654	0.02917376	0.0209541
BP	GO:0010820	positive regu	2/137	16/18670	0.00599654	0.02917376	0.0209541
BP	GO:0033033	negative regu	2/137	16/18670	0.00599654	0.02917376	0.0209541
BP	GO:0006213	pyrimidine nu	3/137	51/18670	0.00622507	0.03019703	0.02168905
BP	GO:0032611	interleukin-1	4/137	101/18670	0.00654329	0.03155614	0.02266524
BP	GO:0050830	defense resp	4/137	101/18670	0.00654329	0.03155614	0.02266524
BP	GO:0032731	positive regu	3/137	52/18670	0.00657119	0.03159885	0.02269591
BP	GO:0010819	regulation of	2/137	17/18670	0.00676357	0.0323365	0.02322573
BP	GO:0043373	CD4-positive,	2/137	17/18670	0.00676357	0.0323365	0.02322573
BP	GO:0050702	interleukin-1	3/137	53/18670	0.00692857	0.0328414	0.02358838
BP	GO:0050704	regulation of	3/137	53/18670	0.00692857	0.0328414	0.02358838

BP	GO:0070228	regulation of	3/137	53/18670	0.00692857	0.0328414	0.02358838
BP	GO:0032652	regulation of	4/137	103/18670	0.00700663	0.03311683	0.0237862
BP	GO:0032663	regulation of	3/137	54/18670	0.00729728	0.0343926	0.02470253
BP	GO:0043011	myeloid den	2/137	18/18670	0.00757265	0.0353888	0.02541805
BP	GO:0070233	negative regu	2/137	18/18670	0.00757265	0.0353888	0.02541805
BP	GO:0090026	positive regu	2/137	18/18670	0.00757265	0.0353888	0.02541805
BP	GO:0043551	regulation of	3/137	55/18670	0.00767743	0.03577768	0.02569737
BP	GO:0002286	T cell activati	4/137	106/18670	0.00774153	0.03597533	0.02583933
BP	GO:0002381	immunoglob	3/137	56/18670	0.00806908	0.03739274	0.02685739
BP	GO:0050708	regulation of	9/137	472/18670	0.00811671	0.03750867	0.02694066
BP	GO:0038095	Fc-epsilon re	5/137	169/18670	0.00820546	0.0378135	0.02715959
BP	GO:0051607	defense resp	6/137	238/18670	0.00829309	0.03811146	0.02737361
BP	GO:0002363	alpha-beta T	2/137	19/18670	0.00842312	0.03838998	0.02757365
BP	GO:0002544	chronic inflar	2/137	19/18670	0.00842312	0.03838998	0.02757365
BP	GO:0002710	negative regu	2/137	19/18670	0.00842312	0.03838998	0.02757365
BP	GO:1903532	positive regu	8/137	399/18670	0.00930194	0.04153893	0.02983539
BP	GO:0032732	positive regu	3/137	59/18670	0.00931381	0.04153893	0.02983539
BP	GO:0002320	lymphoid pro	2/137	20/18670	0.00931434	0.04153893	0.02983539
BP	GO:0002577	regulation of	2/137	20/18670	0.00931434	0.04153893	0.02983539
BP	GO:0032816	positive regu	2/137	20/18670	0.00931434	0.04153893	0.02983539
BP	GO:0033630	positive regu	2/137	20/18670	0.00931434	0.04153893	0.02983539
BP	GO:0043369	CD4-positive	2/137	20/18670	0.00931434	0.04153893	0.02983539
BP	GO:1900017	positive regu	2/137	20/18670	0.00931434	0.04153893	0.02983539
BP	GO:0050868	negative regu	4/137	112/18670	0.0093596	0.04162888	0.0299
BP	GO:0007015	actin filamen	8/137	400/18670	0.00943461	0.04185029	0.03005903
BP	GO:0034113	heterotypic c	3/137	61/18670	0.01020245	0.04473044	0.0321277
BP	GO:0045123	cellular extra	3/137	61/18670	0.01020245	0.04473044	0.0321277
BP	GO:0050701	interleukin-1	3/137	61/18670	0.01020245	0.04473044	0.0321277
BP	GO:0032612	interleukin-1	4/137	115/18670	0.01024524	0.04473044	0.0321277
BP	GO:0001779	natural killer	2/137	21/18670	0.01024567	0.04473044	0.0321277
BP	GO:0035743	CD4-positive,	2/137	21/18670	0.01024567	0.04473044	0.0321277
BP	GO:0019058	viral life cycle	7/137	328/18670	0.01069243	0.04655836	0.03344061
BP	GO:0032496	response to l	7/137	330/18670	0.01102914	0.04777374	0.03431355
BP	GO:0042742	defense resp	7/137	330/18670	0.01102914	0.04777374	0.03431355
BP	GO:0034394	protein locali	3/137	63/18670	0.01113874	0.04795918	0.03444675
BP	GO:0006911	phagocytosis	4/137	118/18670	0.0111835	0.04795918	0.03444675
BP	GO:0051924	regulation of	6/137	254/18670	0.01119693	0.04795918	0.03444675
BP	GO:0006925	inflammatory	2/137	22/18670	0.01121649	0.04795918	0.03444675
BP	GO:0072643	interferon-ga	2/137	22/18670	0.01121649	0.04795918	0.03444675
BP	GO:0002791	regulation of	9/137	500/18670	0.01154669	0.04924413	0.03536966
BP	GO:0043550	regulation of	3/137	64/18670	0.01162489	0.04932402	0.03542704
BP	GO:0046834	lipid phosphc	3/137	64/18670	0.01162489	0.04932402	0.03542704
BP	GO:0030099	myeloid cell (	8/137	416/18670	0.011755	0.04974884	0.03573217
BP	GO:0050854	regulation of	3/137	65/18670	0.0121231	0.05117616	0.03675734
BP	GO:0010499	proteasomal	2/137	23/18670	0.01222618	0.0514803	0.03697579
BP	GO:0042108	positive regu	3/137	67/18670	0.01315592	0.05505443	0.03954292
BP	GO:0051209	release of se	4/137	124/18670	0.01322221	0.05505443	0.03954292
BP	GO:0032897	negative regu	2/137	24/18670	0.01327413	0.05505443	0.03954292
BP	GO:0043302	positive regu	2/137	24/18670	0.01327413	0.05505443	0.03954292
BP	GO:0050857	positive regu	2/137	24/18670	0.01327413	0.05505443	0.03954292

BP	GO:0071677	positive regu	2/137	24/18670	0.01327413	0.05505443	0.03954292
BP	GO:0002237	response to r	7/137	343/18670	0.01340851	0.0554731	0.03984362
BP	GO:0051047	positive regu	8/137	428/18670	0.01375394	0.05676066	0.04076842
BP	GO:0051283	negative regu	4/137	126/18670	0.01395094	0.05743078	0.04124973
BP	GO:0070227	lymphocyte a	3/137	69/18670	0.01423752	0.05796293	0.04163195
BP	GO:0050714	positive regu	6/137	268/18670	0.01427382	0.05796293	0.04163195
BP	GO:0099024	plasma mem	4/137	127/18670	0.01432468	0.05796293	0.04163195
BP	GO:0002507	tolerance ind	2/137	25/18670	0.01435971	0.05796293	0.04163195
BP	GO:0002719	negative regu	2/137	25/18670	0.01435971	0.05796293	0.04163195
BP	GO:0031664	regulation of	2/137	25/18670	0.01435971	0.05796293	0.04163195
BP	GO:0050901	leukocyte tet	2/137	25/18670	0.01435971	0.05796293	0.04163195
BP	GO:0090025	regulation of	2/137	25/18670	0.01435971	0.05796293	0.04163195
BP	GO:0051282	regulation of	4/137	128/18670	0.01470472	0.05921147	0.04252872
BP	GO:0070988	demethylatic	3/137	70/18670	0.0147967	0.0594376	0.04269113
BP	GO:0006816	calcium ion t	8/137	434/18670	0.0148419	0.05947515	0.04271811
BP	GO:1903038	negative regu	4/137	129/18670	0.01509109	0.06032798	0.04333065
BP	GO:0002230	positive regu	2/137	26/18670	0.01548235	0.06130122	0.04402968
BP	GO:0002360	T cell lineage	2/137	26/18670	0.01548235	0.06130122	0.04402968
BP	GO:0051125	regulation of	2/137	26/18670	0.01548235	0.06130122	0.04402968
BP	GO:0072539	T-helper 17 c	2/137	26/18670	0.01548235	0.06130122	0.04402968
BP	GO:0051208	sequestering	4/137	131/18670	0.01588294	0.06273761	0.04506137
BP	GO:0050688	regulation of	3/137	73/18670	0.01654817	0.06520999	0.04683716
BP	GO:0043921	modulation b	2/137	27/18670	0.01664143	0.06526745	0.04687843
BP	GO:0052472	modulation b	2/137	27/18670	0.01664143	0.06526745	0.04687843
BP	GO:0007266	Rho protein s	5/137	203/18670	0.01704352	0.0666868	0.04789789
BP	GO:0010324	membrane ir	4/137	135/18670	0.01754397	0.0684834	0.04918829
BP	GO:0001773	myeloid dend	2/137	28/18670	0.01783638	0.06897564	0.04954185
BP	GO:0035456	response to i	2/137	28/18670	0.01783638	0.06897564	0.04954185
BP	GO:0052312	modulation c	2/137	28/18670	0.01783638	0.06897564	0.04954185
BP	GO:2000108	positive regu	2/137	28/18670	0.01783638	0.06897564	0.04954185
BP	GO:0045454	cell redox ho	3/137	76/18670	0.018411	0.07103222	0.05101899
BP	GO:0070229	negative regu	2/137	29/18670	0.01906661	0.07322107	0.05259113
BP	GO:0072538	T-helper 17 t	2/137	29/18670	0.01906661	0.07322107	0.05259113
BP	GO:0002793	positive regu	6/137	288/18670	0.01962819	0.07520362	0.0540151
BP	GO:0097553	calcium ion t	4/137	140/18670	0.01976759	0.07556321	0.05427338
BP	GO:0001782	B cell homeo	2/137	30/18670	0.02033155	0.07700922	0.05531198
BP	GO:0032743	positive regu	2/137	30/18670	0.02033155	0.07700922	0.05531198
BP	GO:0048873	homeostasis	2/137	30/18670	0.02033155	0.07700922	0.05531198
BP	GO:0050690	regulation of	2/137	30/18670	0.02033155	0.07700922	0.05531198
BP	GO:0050871	positive regu	4/137	142/18670	0.02070355	0.07823961	0.0561957
BP	GO:0070374	positive regu	5/137	215/18670	0.02126271	0.08017007	0.05758226
BP	GO:0002675	positive regu	2/137	31/18670	0.02163063	0.08100501	0.05818195
BP	GO:0002888	positive regu	2/137	31/18670	0.02163063	0.08100501	0.05818195
BP	GO:0043304	regulation of	2/137	31/18670	0.02163063	0.08100501	0.05818195
BP	GO:0001818	negative regu	6/137	296/18670	0.02210892	0.08260668	0.05933236
BP	GO:0002431	Fc receptor n	4/137	145/18670	0.02215791	0.08260668	0.05933236
BP	GO:0033006	regulation of	2/137	32/18670	0.02296329	0.08503595	0.06107718
BP	GO:0070207	protein homo	2/137	32/18670	0.02296329	0.08503595	0.06107718
BP	GO:2000778	positive regu	2/137	32/18670	0.02296329	0.08503595	0.06107718
BP	GO:0060218	hematopoiet	3/137	83/18670	0.02319307	0.08569555	0.06155094

BP	GO:0051092	positive regu	4/137	149/18670	0.02419208	0.08909885	0.06399537
BP	GO:0010591	regulation of	2/137	33/18670	0.02432898	0.08909885	0.06399537
BP	GO:0050718	positive regu	2/137	33/18670	0.02432898	0.08909885	0.06399537
BP	GO:0070232	regulation of	2/137	33/18670	0.02432898	0.08909885	0.06399537
BP	GO:0045066	regulatory T	2/137	34/18670	0.02572714	0.09401174	0.06752406
BP	GO:0070838	divalent met	8/137	483/18670	0.026187	0.09548185	0.06857996
BP	GO:0030168	platelet activ	4/137	153/18670	0.02633597	0.09581441	0.06881882
BP	GO:1903725	regulation of	3/137	88/18670	0.02698272	0.09795258	0.07035457
BP	GO:0002701	negative regu	2/137	35/18670	0.02715724	0.09815656	0.07050108
BP	GO:0042092	type 2 immu	2/137	35/18670	0.02715724	0.09815656	0.07050108
CC	GO:0001772	immunologic	6/143	36/19717	2.1327E-07	3.647E-05	3.0981E-05
CC	GO:0045335	phagocytic ve	9/143	132/19717	5.1346E-07	4.3901E-05	3.7293E-05
CC	GO:0042629	mast cell gra	4/143	22/19717	1.7531E-05	0.00099927	0.00084887
CC	GO:0030139	endocytic ve	10/143	303/19717	7.477E-05	0.00319641	0.00271532
CC	GO:0009897	external side	11/143	393/19717	0.00014121	0.00454423	0.00386029
CC	GO:0043020	NADPH oxida	3/143	15/19717	0.00015945	0.00454423	0.00386029
CC	GO:0001891	phagocytic cu	3/143	27/19717	0.00096173	0.01908956	0.01621643
CC	GO:0005942	phosphatidyl	3/143	27/19717	0.00096173	0.01908956	0.01621643
CC	GO:0101003	ficolin-1-rich	4/143	61/19717	0.00100471	0.01908956	0.01621643
CC	GO:0070820	tertiary granu	6/143	164/19717	0.00126351	0.01992764	0.01692838
CC	GO:0005884	actin filamen	5/143	111/19717	0.00129507	0.01992764	0.01692838
CC	GO:0098802	plasma mem	8/143	295/19717	0.00142128	0.01992764	0.01692838
CC	GO:0030667	secretory gra	8/143	298/19717	0.00151497	0.01992764	0.01692838
CC	GO:0030670	phagocytic ve	4/143	76/19717	0.00227166	0.02670792	0.02268817
CC	GO:0042101	T cell recepto	5/143	127/19717	0.0023428	0.02670792	0.02268817
CC	GO:0019774	proteasome	2/143	11/19717	0.00275226	0.02941473	0.02498758
CC	GO:0005767	secondary lys	2/143	14/19717	0.00448927	0.04515678	0.03836033
CC	GO:0019898	extrinsic com	7/143	295/19717	0.00580582	0.05515525	0.04685396
CC	GO:0030666	endocytic ve	5/143	167/19717	0.00746554	0.06718985	0.05707725
CC	GO:0005839	proteasome	2/143	20/19717	0.00911038	0.07789374	0.06617012
CC	GO:0098562	cytoplasmic s	5/143	178/19717	0.00968143	0.07883454	0.06696932
MF	GO:0016176	superoxide-g	3/134	10/17697	4.8998E-05	0.00441655	0.00347297
MF	GO:0005164	tumor necros	4/134	31/17697	8.4378E-05	0.00441655	0.00347297
MF	GO:0004126	cytidine dear	3/134	12/17697	8.8837E-05	0.00441655	0.00347297
MF	GO:0004896	cytokine rece	6/134	96/17697	8.9368E-05	0.00441655	0.00347297
MF	GO:0019239	deaminase a	4/134	32/17697	9.5869E-05	0.00441655	0.00347297
MF	GO:0030695	GTPase regul	10/134	304/17697	0.00010772	0.00441655	0.00347297
MF	GO:0016814	hydrolase act	4/134	34/17697	0.0001222	0.00441655	0.00347297
MF	GO:0005125	cytokine acti	8/134	220/17697	0.00027366	0.00764154	0.00600895
MF	GO:0060589	nucleoside-tr	10/134	344/17697	0.0002925	0.00764154	0.00600895
MF	GO:0048020	CCR chemoki	4/134	43/17697	0.00030849	0.00764154	0.00600895
MF	GO:0005126	cytokine rece	9/134	286/17697	0.00033224	0.00764154	0.00600895
MF	GO:0004715	non-membra	4/134	46/17697	0.00040082	0.00780056	0.00613399
MF	GO:0032813	tumor necros	4/134	46/17697	0.00040082	0.00780056	0.00613399
MF	GO:0042288	MHC class I p	3/134	20/17697	0.00044036	0.00795795	0.00625776
MF	GO:0008009	chemokine a	4/134	49/17697	0.00051137	0.00862515	0.00678242
MF	GO:0005096	GTPase activ	8/134	273/17697	0.00113071	0.01787934	0.01405948
MF	GO:1901981	phosphatidyl	6/134	161/17697	0.0014228	0.01976004	0.01553837
MF	GO:0043325	phosphatidyl	3/134	30/17697	0.00148396	0.01976004	0.01553837
MF	GO:0043548	phosphatidyl	3/134	30/17697	0.00148396	0.01976004	0.01553837

MF	GO:0042379	chemokine re	4/134	66/17697	0.00157536	0.01992834	0.01567071
MF	GO:0019955	cytokine binc	5/134	128/17697	0.00290525	0.03292273	0.0258889
MF	GO:0019864	IgG binding	2/134	11/17697	0.00299298	0.03292273	0.0258889
MF	GO:0035004	phosphatidyl	2/134	11/17697	0.00299298	0.03292273	0.0258889
MF	GO:0005547	phosphatidyl	3/134	39/17697	0.00317875	0.03348786	0.02633329
MF	GO:0042287	MHC protein	3/134	40/17697	0.00341763	0.03348786	0.02633329
MF	GO:0004713	protein tyros	5/134	134/17697	0.00353685	0.03348786	0.02633329
MF	GO:0016175	superoxide-g	2/134	12/17697	0.0035738	0.03348786	0.02633329
MF	GO:0046935	1-phosphatic	2/134	14/17697	0.00487893	0.0440846	0.03466607
MF	GO:0035014	phosphatidyl	2/134	17/17697	0.00718416	0.06058642	0.04764233
MF	GO:0050664	oxidoreducta	2/134	17/17697	0.00718416	0.06058642	0.04764233
MF	GO:0004298	threonine-ty	2/134	21/17697	0.01087627	0.08384232	0.06592968
MF	GO:0070003	threonine-ty	2/134	21/17697	0.01087627	0.08384232	0.06592968
MF	GO:0035091	phosphatidyl	6/134	245/17697	0.01093595	0.08384232	0.06592968
MF	GO:0016493	C-C chemokir	2/134	23/17697	0.0129748	0.09378925	0.07375148
MF	GO:0046625	sphingolipid	2/134	23/17697	0.0129748	0.09378925	0.07375148
MF	GO:0019865	immunoglob	2/134	24/17697	0.0140848	0.09503585	0.07473175
MF	GO:0019957	C-C chemokir	2/134	24/17697	0.0140848	0.09503585	0.07473175
MF	GO:0016810	hydrolase act	4/134	123/17697	0.01427416	0.09503585	0.07473175
MF	GO:0048365	Rac GTPase b	3/134	69/17697	0.01546481	0.09892899	0.07779313
MF	GO:0001637	G protein-cou	2/134	26/17697	0.016423	0.09892899	0.07779313
MF	GO:0004435	phosphatidyl	2/134	26/17697	0.016423	0.09892899	0.07779313
MF	GO:0004950	chemokine re	2/134	26/17697	0.016423	0.09892899	0.07779313
KEGG	hsa04060	Cytokine-cyto	16/84	295/8076	4.5304E-08	3.115E-06	2.5813E-06
KEGG	hsa04061	Viral protein	10/84	100/8076	6.9446E-08	3.115E-06	2.5813E-06
KEGG	hsa04380	Osteoclast di	11/84	128/8076	7.1684E-08	3.115E-06	2.5813E-06
KEGG	hsa04062	Chemokine s	13/84	192/8076	7.3675E-08	3.115E-06	2.5813E-06
KEGG	hsa04650	Natural killer	11/84	131/8076	9.1138E-08	3.115E-06	2.5813E-06
KEGG	hsa05340	Primary imm	7/84	38/8076	9.9414E-08	3.115E-06	2.5813E-06
KEGG	hsa05235	PD-L1 expres	8/84	89/8076	3.4838E-06	9.3564E-05	7.7534E-05
KEGG	hsa05142	Chagas disea	8/84	102/8076	9.6974E-06	0.00022789	0.00018884
KEGG	hsa04064	NF-kappa B s	8/84	104/8076	1.12E-05	0.00023396	0.00019388
KEGG	hsa04658	Th1 and Th2	7/84	92/8076	4.3916E-05	0.00082563	0.00068417
KEGG	hsa05170	Human immu	10/84	212/8076	6.3663E-05	0.00108805	0.00090163
KEGG	hsa04659	Th17 cell diff	7/84	107/8076	0.00011532	0.00180663	0.0014971
KEGG	hsa04672	Intestinal imr	5/84	49/8076	0.00014374	0.00207871	0.00172256
KEGG	hsa04623	Cytosolic DN.	5/84	63/8076	0.00047282	0.00634935	0.0052615
KEGG	hsa04660	T cell recepto	6/84	104/8076	0.00071181	0.00892129	0.00739278
KEGG	hsa04662	B cell recepto	5/84	82/8076	0.00157335	0.01848691	0.0153195
KEGG	hsa04611	Platelet activ	6/84	124/8076	0.00177641	0.01964499	0.01627916
KEGG	hsa05166	Human T-cell	8/84	219/8076	0.00190943	0.01994297	0.01652609
KEGG	hsa05163	Human cytor	8/84	225/8076	0.00226201	0.02238198	0.01854722
KEGG	hsa05135	Yersinia infec	6/84	137/8076	0.00293777	0.02761505	0.02288369
KEGG	hsa04666	Fc gamma R-	5/84	97/8076	0.00329167	0.0294683	0.02441942
KEGG	hsa04640	Hematopoiet	5/84	99/8076	0.00359463	0.03071774	0.02545479
KEGG	hsa04145	Phagosome	6/84	152/8076	0.00489854	0.04004025	0.03318005
KEGG	hsa04664	Fc epsilon RI	4/84	68/8076	0.00535163	0.04192111	0.03473865
KEGG	hsa05152	Tuberculosis	6/84	180/8076	0.01091086	0.08093454	0.06706781
KEGG	hsa04621	NOD-like rec	6/84	181/8076	0.01119307	0.08093454	0.06706781

geneID	Count
AIF1/CD3D/C	34
AIF1/BTK/CD	29
AIF1/CD3E/C	25
AIF1/CD3E/C	23
AIF1/BTK/CD	25
AIF1/BTK/CD	23
AIF1/BTK/CD	22
AIF1/CD3E/C	19
AIF1/BTK/CD	24
AIF1/BTK/CD	21
AIF1/BTK/CD	21
AIF1/BTK/CD	19
AIF1/BTK/CD	19
AIF1/CD3E/C	19
AIF1/BTK/CD	19
AIF1/CD3E/C	21
AIF1/CD3E/C	19
AIF1/CD3E/C	22
AIF1/CD3E/C	13
AIF1/CXCR5/	24
AIF1/CD3E/C	16
AIF1/CD3E/C	15
BTK/C1QA/C	19
AIF1/CD3E/C	13
AIF1/CD3E/C	13
CD3D/CD3E/	16
AIF1/CD3E/C	13
AIF1/CXCR3/	12
BTK/CD3D/C	18
AIF1/CD3E/C	19
BTK/C1QA/C	20
AIF1/CD3E/C	20
BTK/CD3D/C	20
BTK/CD3D/C	14
TNFRSF17/C	16
BTK/HLA-F/T	13
TNFRSF17/C	16
BTK/C1QA/C	17
BTK/CD3D/C	16
BTK/HLA-F/T	13
CXCR5/BTK/	15
AIF1/CXCR5/	13
AIF1/CXCR3/	8
BTK/FCER1G/	12
TNFRSF17/C	11
AIF1/RHOG/	14
BTK/FCER1G/	12
CD3E/FUT7/	10
BTK/HLA-F/T	10

BTK/FCER1G/	10
TNFRSF17/FC	8
HLA-F/PIK3CI	8
CD52/NCF4/I	6
BTK/FCER1G/	7
BTK/CD48/FC	10
BTK/FCER1G/	16
CXCR3/PIK3C	7
AIF1/BTK/CD	13
PIK3CD/CCL4	4
BTK/FCER1G/	8
BTK/FCER1G/	6
GZMM/HLA-I	8
BTK/FCER1G/	6
BTK/FCER1G/	12
BTK/FCER1G/	6
RHOG/ARHG	14
FCER1G/FCG	7
LSP1/TYROBI	6
RHOG/ARHG	15
AIF1/CD247/	13
CD3E/LCK/TN	6
CD3E/LCK/TN	6
GZMM/HLA-I	9
CD3D/CD3E/	5
BTK/FCER1G/	13
HLA-F/IL18R/	6
BTK/C1QA/C	10
CD3E/ZAP70/	7
BTK/FCER1G/	14
BTK/FCER1G/	8
AIF1/CCL4/C	6
FCER1G/FCG	7
AIF1/CCL4/C	5
AIF1/FCGR1A	9
HLA-F/IL18R/	6
BTK/FCER1G/	7
BTK/FCER1G/	7
FCER1G/PIK3	7
BTK/FCER1G/	8
BTK/FCER1G/	7
CD70/GZMM	7
AIF1/CXCR3/	9
BTK/TYROBP/	6
BTK/TYROBP/	6
AIF1/FCGR1A	9
BTK/FCER1G/	6
RHOG/FCER1	14
FCGR1A/HLA	6
CD3E/LTA/IL1	7

AIF1/RAC2/C	7
BTK/TYROBP/	6
CD3D/CD3E/	5
FCER1G/PIK3	7
FCER1G/PIK3	7
BTK/ZAP70/T	8
BTK/FCER1G/	6
ARHGDIB/ISG	8
CCL4/CCL5/X	4
AIF1/RAC2/C	6
AIF1/RAC2/C	7
FCER1G/FCG	9
CXCR5/CXCR	6
CXCR3/PIK3C	4
FCER1G/HLA-	8
FLT3/PIK3CD	8
CD3E/CCL5/F	3
FLT3/PIK3CD	8
CD3E/ZAP70/	4
BTK/TYROBP/	4
BTK/ZAP70/T	6
AIF1/TNFRSF	9
FCER1G/HLA-	6
BTK/CD70/TY	6
ISG20/CCL5/	5
BTK/CD247/F	9
AIF1/CCL5/X	4
BTK/FCER1G/	7
ZAP70/TESPA	7
BTK/CD3E/FC	11
CXCR5/CXCR	6
CXCR5/CXCR	6
BTK/FCER1G/	3
APOBEC3G/A	3
APOBEC3G/A	3
APOBEC3G/A	3
APOBEC3G/A	3
FCER1G/PIK3	7
BTK/CD3E/FC	11
BTK/TYROBP/	5
HLA-F/CORO	4
TNFRSF17/FC	9
ISG20/CCL4/	6
TNFRSF17/TC	5
CD3E/CD247	5
BTK/TYROBP/	7
CD3E/FCER1G	4
CD3E/ZAP70/	4
FUT7/ZAP70/	6
APOBEC3G/A	3

CD3D/CD3E/	3
FLT3/PIK3CD	7
AIF1/CCL4/C	5
CD3E/LTA/KL	5
APOBEC3G/A	3
APOBEC3G/A	3
FCER1G/PIK3	8
BTK/FCER1G,	4
BTK/FCER1G,	4
AIF1/FCER1G	8
FCER1G/IL2R	5
BTK/TYROBP,	9
RHOG/ARHG	10
ARHGDIB/ISG	11
BTK/TYROBP,	3
CD3E/CCL5/F	3
HLA-F/IL18RA	4
BTK/C1QA/C	8
CXCR5/BTK/C	8
RHOG/FCER1	12
APOBEC3G/A	3
CXCR5/LTA/L	3
FCER1G/RAC	4
FLT3/LILRB2/	4
RHOG/FCER1	12
HLA-F/TYROE	5
APOBEC3G/A	3
HLA-F/IL18RA	4
AIF1/CCL5/X	4
FLT3/PIK3CD	6
FCER1G/FCG	7
RHOG/FCER1	12
CD3E/ZAP70,	3
CD247/FCER1	3
BTK/CD6/FCE	4
HLA-F/IL18RA	4
FCER1G/HLA-	4
GBP2/PSMB9	7
ZAP70/TESPA	5
BTK/FCER1G,	5
FCER1G/FCG	7
ISG20/CCL5/	5
AIF1/C1QA/T	4
AIF1/C1QA/T	4
BTK/TYROBP,	7
BTK/TYROBP,	7
FLT3/PIK3CD	5
BTK/CD6/FCE	3
APOBEC3G/A	3
CD3D/CD3E/	3

CCL4/CCL5/C	3
CCL4/CCL5/X	3
AIF1/RAC2/C	6
FCER1G/FCG	7
AIF1/CCL4/C(	5
BTK/FCER1G,	4
BTK/FCER1G,	4
CD3E/FCGR1	7
BTK/TCIRG1/	7
AIF1/TNFRSF	6
FCER1G/TYR(	4
FUT7/TCIRG1	5
BTK/FCER1G,	9
APOBEC3G/A	3
APOBEC3G/A	3
CD3E/FCER1(	3
APOBEC3G/A	3
FCER1G/HLA-	4
ISG20/CCL5/,	5
GBP2/HLA-F/	5
GBP2/HLA-F/	5
ISG20/CCL4/(	9
BTK/FCER1G,	3
GBP2/BATF/I	3
APOBEC3G/A	3
AIF1/FCER1G	5
BTK/FCER1G,	4
FCER1G/TYR(	4
FCGR1A/CCL!	4
HLA-F/TYROE	4
BTK/PSMB9/	11
HLA-F/TYROE	5
FCER1G/IL2R	5
GBP2/BATF/I	3
CCL4/CCL5/C	3
GBP2/HLA-F/	5
GBP2/PSMB9	7
CD6/CCL5/LY	4
AIF1/C1QA/T	4
APOBEC3G/A	3
CD3E/LTA/KL	5
BTK/C1QA/C	11
AIF1/TNFRSF	7
BTK/CD6/FCE	6
FCER1G/ITGE	5
CD3E/FCER1(	3
BTK/FCER1G,	5
APOBEC3G/A	3
CD3E/ZAP70,	4
AIF1/RAC2/C	7

ZAP70/TBX2[illegible]	4
AIF1/FCER1G	6
FCER1G/PIK3	3
HLA-F/IL18R[illegible]	3
FLT3/PSMB9[illegible]	6
FCER1G/PSM	5
HLA-F/TBX21	3
FCER1G/BAT	4
HLA-F/TYROE	4
CD3E/ITGB7/	4
BTK/FCER1G/	8
HLA-F/TYROE	3
APOBEC3G/A	3
APOBEC3G/A	3
IL2RG/CORO	3
FCER1G/PSM	5
AIF1/RHOG/[illegible]	10
HLA-F/WAS/[illegible]	4
CD3D/CD3E/	4
HLA-F/IL18R[illegible]	3
FCER1G/PIK3	3
BTK/CD6/CCI	7
BTK/CD6/FCE	4
APOBEC3G/A	3
AIF1/RHOG/[illegible]	3
IL2RG/CORO	3
NCF4/TYROB	4
CXCR5/CD52[illegible]	10
ISG20/CCL5/[illegible]	5
APOBEC3G/A	3
FUT7/BATF/T	4
AIF1/C1QA/T	4
FCER1G/SASI	3
CXCR5/CD52[illegible]	8
CXCR5/CD52[illegible]	10
BTK/LCK/PIK[illegible]	5
CD3E/LILRB2	2
CD3E/ZAP70	2
IL18RAP/IL1[illegible]	2
C1QA/C1QB	2
CD3E/FCGR1	7
LCK/TESPA1/	3
APOBEC3G/A	3
CD247/FCER[illegible]	3
PIK3CD/RAC[illegible]	2
CD3E/ZAP70	2
CCL4/CCL5	2
RAC2/ZAP70	2
BATF/TBX21	2
CST7/CARD1[illegible]	2

CST7/CARD1(	2
PIK3CD/RAC2	2
CXCR5/CD52,	10
AIM2/LY86/D	5
FCER1G/TYR(	4
CD3E/CCL4/Z	3
APOBEC3G/A	3
BTK/FCER1G	2
CD300LF/MII	2
IL2RG/IL15RA	2
IL2RG/IL15RA	2
CD247/FCER1	5
FCGR1A/CCL5	4
FCER1G/TYR(	4
CD6/CCL5/LY	6
TYROBP/AIM	4
ISG20/CCL4/(	6
HLA-F/TNFRS	3
LCK/TESPA1	2
IL2RG/IL15RA	2
CD3E/CCL5/F	3
AIM2/CARD1	3
CD6/CCL5/LY	6
ISG20/CCL5/A	5
CXCR5/CD52,	8
FCER1G/CCL5	3
RHOG/FMNL	5
AIF1/NCF4/R	7
CXCR5/BTK/(	9
AIF1/C1QA/T	4
AIF1/FCER1G	5
FCER1G/IL2R	5
FCER1G/TYR(	4
BTK/C1QA/C	6
CD3E/CCL4/Z	4
APOBEC3G/A	3
PIK3CD/SLA2	3
ISG20/CCL4/(	6
BATF/TBX21	2
CD3E/LILRB2	2
CCL5/XCL2	2
FCER1G/CCL5	2
APOBEC3G/A	3
TYROBP/AIM	4
GBP2/LTA/TN	4
TYROBP/AIM	3
CCL5/XCL2	2
BATF/TBX21	2
AIM2/CARD1	3
AIM2/CARD1	3

BTK/CCL5/PT	3
TYROBP/AIM	4
CD3E/TBX21,	3
BATF/BATF2	2
CCL5/PTCRA	2
AIF1/CCL5	2
FLT3/SLA2/P	3
FCER1G/BAT	4
BTK/BATF/TE	3
AIF1/CCL5/TI	9
BTK/FCER1G,	5
ISG20/AIM2/	6
BATF/TBX21	2
LTA/CCL5	2
HLA-F/TBX21	2
AIF1/FCER1G	8
TYROBP/AIM	3
FLT3/BATF	2
WAS/LILRB2	2
HLA-F/TYROE	2
CD3E/CCL5	2
BATF/TBX21	2
CD6/GPSM3	2
TNFRSF14/LII	4
AIF1/RHOG/I	8
ITGB7/LCK/LI	3
ITGB7/PIK3C	3
AIM2/CARD1	3
TYROBP/AIM	4
PIK3CD/ZNF6	2
IL18RAP/TBX	2
ISG20/ITGB7,	7
CD6/LTA/CCL	7
FCER1G/GBP	7
CD247/FCER1	3
AIF1/FCER1G	4
CCL4/CCL5/L	6
PIK3CD/CCL5	2
TCIRG1/CD24	2
AIF1/CCL5/TI	9
FLT3/SLA2/PI	3
PIK3CD/SLA2	3
FCER1G/PIK3	8
LCK/TESPA1/	3
PSMB9/PSMI	2
CD3E/LTB/TY	3
LCK/PLCB2/C	4
CCL4/CCL5	2
FCER1G/HLA-	2
LCK/TESPA1	2

AIF1/CCL5	2
CD6/LTA/CCL	7
AIF1/FCER1G	8
LCK/PLCB2/C	4
BTK/CCL5/PT	3
AIF1/TNFRSF	6
AIF1/FCER1G	4
CD3E/LILRB2	2
HLA-F/TBX21	2
LY86/CARD1(	2
ITGB7/SELL	2
AIF1/CCL5	2
LCK/PLCB2/C	4
APOBEC3G/A	3
LCK/PLCB2/C	8
TNFRSF14/LI	4
AIM2/APOBE	2
BATF/TBX21	2
WAS/CORO1	2
BATF/TBX21	2
LCK/PLCB2/C	4
AIM2/DDX60	3
CCL4/CCL5	2
CCL4/CCL5	2
RHOG/ARHG	5
AIF1/FCER1G	4
BATF/BATF2	2
AIM2/XAF1	2
CCL4/CCL5	2
PIK3CD/CCL5	2
NCF4/RAC2/I	3
CCL5/PTCRA	2
BATF/TBX21	2
AIF1/TNFRSF	6
LCK/PLCB2/C	4
TNFSF13B/TN	2
CD3E/SASH3	2
CORO1A/SAS	2
CD247/LCK	2
BTK/TNFSF13	4
FLT3/CCL4/C	5
BTK/FCER1G	2
BTK/FCER1G	2
FCER1G/RAC	2
BTK/HLA-F/T	6
CD247/FCER1	4
FCER1G/RAC	2
CD247/MLKL	2
AIF1/LILRA5	2
PSMB9/PSMI	3

BTK/IL18RAP	4
RAC2/WAS	2
AIM2/LILRA5	2
CCL5/PTCRA	2
FUT7/LILRB2	2
LCK/PLCB2/C	8
FCER1G/LCK/	4
FLT3/SLA2/P	3
HLA-F/TBX21	2
BATF/TBX21	2
CD3E/CD6/G	6
FMNL1/HLA-	9
BTK/PIK3CD/	4
FMNL1/FCGF	10
CXCR5/CD3D	11
NCF4/NCF1/I	3
AIF1/ARHGAI	3
PIK3CD/SLA2	3
FCER1G/LILR	4
FCER1G/LAIR	6
AIF1/RAC2/V	5
CD3D/CD3E/	8
RHOG/FCER1	8
HLA-F/RAC2/	4
CD3D/CD3E/	5
PSMB9/PSMI	2
NCF4/NCF1	2
GNGT2/LCK/	7
FCGR1A/HLA	5
PSMB9/PSMI	2
GNGT2/LCK/	5
NCF4/NCF1/I	3
CD70/LTA/LT	4
APOBEC3G/A	3
CXCR5/FLT3/	6
APOBEC3G/Z	4
ARHGDIB/W/	10
APOBEC3G/Z	4
CD70/LTA/LT	8
ARHGDIB/W/	10
CCL4/CCL5/X	4
CD70/LTA/LT	9
BTK/LCK/MA	4
CD70/LTA/LT	4
LILRB2/KLRK:	3
CCL4/CCL5/X	4
ARHGDIB/AC	8
BTK/NCF4/AI	6
ADAP2/NCF1	3
LCK/HCST/CC	3

CCL4/CCL5/X	4
CXCR5/CXCR	5
FCER1G/FCG	2
PIK3CD/PIK3I	2
BTK/ADAP2/	3
LILRB2/KLRK	3
BTK/FLT3/LC	5
NCF1/NCF1C	2
SLA2/PIK3R6	2
SLA2/PIK3R6	2
NCF1/NCF1C	2
PSMB9/PSMI	2
PSMB9/PSMI	2
BTK/NCF4/AI	6
CXCR5/CXCR	2
SELL/CD300L	2
FCER1G/FCG	2
CXCR5/CXCR	2
APOBEC3G/Z	4
ARHGDIB/FM	3
CXCR5/CXCR	2
PLCB2/CCL5	2
CXCR5/CXCR	2
TNFRSF17/C)	16
CXCR5/CXCR	10
BTK/FCGR1A,	11
CXCR5/GNGT	13
CD247/CD48	11
BTK/CD3D/CI	7
CD3D/CD3E/	8
C1QA/C1QB/	8
BTK/LCK/LTA	8
CD3D/CD3E/	7
CD3D/CD3E/	10
CD3D/CD3E/	7
TNFRSF17/IL	5
CCL4/CCL5/A	5
CD3D/CD3E/	6
BTK/PIK3CD/	5
BTK/FCER1G,	6
CD3D/CD3E/	8
GNGT2/HLA-	8
RHOG/LCK/P	6
FCGR1A/PIK3	5
CD3D/CD3E/	5
FCGR1A/HLA	6
BTK/FCER1G,	4
FCER1G/FCG	6
GBP2/PLCB2,	6
