# Supplementary material for: A Comprehensive Analysis of Interferon Regulatory Factor Expression: Correlation with Immune Cell Infiltration and Patient Prognosis in Endometrial Carcinoma
Source: Biomed Res Int. 2022 Aug 8;2022:7948898. doi: 10.1155/2022/7948898 (PMC9381850; doi:10.1155/2022/7948898)
Supplement: Supplementary 3 — Supplementary Table 3: Gene Ontology (GO) and Kyoto Encyclopedia of Genes and Genomes (KEGG) analyses of functions of IRFs genes. [file 7948898.f3.pdf]

ONTOLOGY	ID	Description	GeneRatio	BgRatio	pvalue	p.adjust	qvalue
BP	GO:0060333	interferon-ga	9/9	91/18670	1.0339E-21	1.2348E-19	4.1334E-20
BP	GO:0060337	type I interfe	9/9	95/18670	1.55E-21	1.2348E-19	4.1334E-20
BP	GO:0071357	cellular respc	9/9	95/18670	1.55E-21	1.2348E-19	4.1334E-20
BP	GO:0034340	response to t	9/9	99/18670	2.2835E-21	1.3644E-19	4.5671E-20
BP	GO:0071346	cellular respc	9/9	180/18670	5.8848E-19	2.8129E-17	9.4157E-18
BP	GO:0034341	response to i	9/9	199/18670	1.4808E-18	5.8987E-17	1.9745E-17
BP	GO:0051607	defense resp	6/9	238/18670	3.2786E-10	1.1194E-08	3.7469E-09
BP	GO:0032728	positive regu	4/9	30/18670	6.785E-10	2.027E-08	6.785E-09
BP	GO:0009615	response to v	6/9	326/18670	2.1761E-09	5.7789E-08	1.9344E-08
BP	GO:0032648	regulation of	4/9	48/18670	4.7989E-09	1.1469E-07	3.8391E-08
BP	GO:0032608	interferon-be	4/9	50/18670	5.6774E-09	1.2336E-07	4.1291E-08
BP	GO:0001819	positive regu	6/9	464/18670	1.7992E-08	3.5835E-07	1.1995E-07
BP	GO:0032481	positive regu	4/9	77/18670	3.3169E-08	6.0979E-07	2.0411E-07
BP	GO:0032727	positive regu	3/9	22/18670	1.1874E-07	2.0271E-06	6.7852E-07
BP	GO:0042089	cytokine bios	4/9	123/18670	2.2033E-07	3.2532E-06	1.0889E-06
BP	GO:0042107	cytokine met	4/9	124/18670	2.2762E-07	3.2532E-06	1.0889E-06
BP	GO:0032479	regulation of	4/9	126/18670	2.4275E-07	3.2532E-06	1.0889E-06
BP	GO:0032647	regulation of	3/9	28/18670	2.5223E-07	3.2532E-06	1.0889E-06
BP	GO:0032606	type I interfe	4/9	128/18670	2.5862E-07	3.2532E-06	1.0889E-06
BP	GO:0032607	interferon-al	3/9	30/18670	3.1244E-07	3.7336E-06	1.2498E-06
BP	GO:0002224	toll-like rece	4/9	146/18670	4.3863E-07	4.9921E-06	1.671E-06
BP	GO:0032735	positive regu	3/9	35/18670	5.0307E-07	5.4651E-06	1.8293E-06
BP	GO:0002221	pattern reco	4/9	197/18670	1.4537E-06	1.5106E-05	5.0562E-06
BP	GO:0032655	regulation of	3/9	54/18670	1.8978E-06	1.8899E-05	6.3259E-06
BP	GO:0032615	interleukin-1	3/9	56/18670	2.1199E-06	2.0266E-05	6.7836E-06
BP	GO:0034121	regulation of	3/9	70/18670	4.1721E-06	3.8351E-05	1.2837E-05
BP	GO:0002758	innate immu	4/9	298/18670	7.5251E-06	6.6611E-05	2.2297E-05
BP	GO:0039530	MDA-5 signa	2/9	10/18670	9.2771E-06	7.9186E-05	2.6506E-05
BP	GO:0002218	activation of	4/9	319/18670	9.8495E-06	8.1173E-05	2.7171E-05
BP	GO:0045351	type I interfe	2/9	11/18670	1.1336E-05	9.0309E-05	3.0229E-05
BP	GO:0071888	macrophage	2/9	12/18670	1.36E-05	0.00010485	3.5096E-05
BP	GO:0060340	positive regu	2/9	13/18670	1.6068E-05	0.00012001	4.0171E-05
BP	GO:0045089	positive regu	4/9	381/18670	1.9836E-05	0.00014366	4.8086E-05
BP	GO:0045088	regulation of	4/9	452/18670	3.8786E-05	0.00027264	9.1261E-05
BP	GO:0006925	inflammatory	2/9	22/18670	4.748E-05	0.00032422	0.00010852
BP	GO:0042832	defense resp	2/9	24/18670	5.6701E-05	0.00037643	0.000126
BP	GO:0001562	response to p	2/9	25/18670	6.1616E-05	0.000398	0.00013322
BP	GO:0039528	cytoplasmic p	2/9	27/18670	7.2054E-05	0.00045318	0.00015169
BP	GO:0035666	TRIF-depend	2/9	29/18670	8.3303E-05	0.0005105	0.00017088
BP	GO:0002756	MyD88-indep	2/9	33/18670	0.00010823	0.00063088	0.00021117
BP	GO:0033028	myeloid cell a	2/9	33/18670	0.00010823	0.00063088	0.00021117
BP	GO:0060338	regulation of	2/9	35/18670	0.0001219	0.00069366	0.00023219
BP	GO:0002755	MyD88-depe	2/9	36/18670	0.00012904	0.00071721	0.00024007
BP	GO:0044764	multi-organis	2/9	48/18670	0.00023035	0.0012512	0.00041881
BP	GO:1902105	regulation of	3/9	272/18670	0.00024074	0.00127861	0.00042798
BP	GO:0001961	positive regu	2/9	50/18670	0.00025003	0.00129906	0.00043483
BP	GO:0098586	cellular respc	2/9	56/18670	0.00031385	0.00159596	0.00053421
BP	GO:0060760	positive regu	2/9	57/18670	0.00032518	0.00161914	0.00054197
BP	GO:0002753	cytoplasmic p	2/9	62/18670	0.00038481	0.00187691	0.00062826

BP	GO:0042108	positive regu	2/9	67/18670	0.00044936	0.00214795	0.00071898
BP	GO:0002237	response to r	3/9	343/18670	0.00047559	0.00222873	0.00074602
BP	GO:0030099	myeloid cell d	3/9	416/18670	0.00083479	0.00383681	0.00128429
BP	GO:0046634	regulation of	2/9	93/18670	0.00086382	0.00389534	0.00130388
BP	GO:0046632	alpha-beta T	2/9	101/18670	0.00101766	0.00450411	0.00150765
BP	GO:0071887	leukocyte ap	2/9	104/18670	0.00107852	0.00468665	0.00156875
BP	GO:1903706	regulation of	3/9	475/18670	0.00122607	0.00523271	0.00175154
BP	GO:0042035	regulation of	2/9	114/18670	0.00129376	0.00542471	0.0018158
BP	GO:0046631	alpha-beta T	2/9	138/18670	0.00188739	0.00775519	0.00259588
BP	GO:0045580	regulation of	2/9	139/18670	0.00191446	0.00775519	0.00259588
BP	GO:0002819	regulation of	2/9	160/18670	0.00252573	0.01006083	0.00336764
BP	GO:0045619	regulation of	2/9	169/18670	0.00281247	0.01101933	0.00368848
BP	GO:0001959	regulation of	2/9	177/18670	0.00307969	0.01187172	0.0039738
BP	GO:0060759	regulation of	2/9	190/18670	0.00353853	0.01342395	0.00449337
BP	GO:0002573	myeloid leuk	2/9	204/18670	0.00406641	0.01509541	0.00505286
BP	GO:0071222	cellular respo	2/9	205/18670	0.00410545	0.01509541	0.00505286
BP	GO:0071219	cellular respo	2/9	212/18670	0.00438362	0.01587403	0.00531348
BP	GO:2000109	regulation of	1/9	10/18670	0.00481128	0.01716263	0.00574481
BP	GO:0019042	viral latency	1/9	11/18670	0.00529128	0.01832775	0.00613481
BP	GO:0032494	response to p	1/9	11/18670	0.00529128	0.01832775	0.00613481
BP	GO:0071216	cellular respo	2/9	236/18670	0.00540237	0.0183356	0.00613744
BP	GO:0007050	cell cycle arre	2/9	237/18670	0.00544698	0.0183356	0.00613744
BP	GO:0030217	T cell differer	2/9	240/18670	0.00558185	0.01852864	0.00620205
BP	GO:0072540	T-helper 17 c	1/9	12/18670	0.00577106	0.01889431	0.00632445
BP	GO:0045086	positive regu	1/9	13/18670	0.00625065	0.0201879	0.00675746
BP	GO:0043374	CD8-positive,	1/9	14/18670	0.00673003	0.02144635	0.00717869
BP	GO:0032736	positive regu	1/9	15/18670	0.0072092	0.0223766	0.00749007
BP	GO:2001185	regulation of	1/9	15/18670	0.0072092	0.0223766	0.00749007
BP	GO:0030522	intracellular r	2/9	280/18670	0.00752625	0.02296839	0.00768816
BP	GO:0002295	T-helper cell	1/9	16/18670	0.00768816	0.02296839	0.00768816
BP	GO:0033033	negative regu	1/9	16/18670	0.00768816	0.02296839	0.00768816
BP	GO:0043373	CD4-positive,	1/9	17/18670	0.00816692	0.02351681	0.00787173
BP	GO:0060644	mammary gla	1/9	17/18670	0.00816692	0.02351681	0.00787173
BP	GO:0071360	cellular respo	1/9	17/18670	0.00816692	0.02351681	0.00787173
BP	GO:0043011	myeloid dend	1/9	18/18670	0.00864548	0.02459845	0.00823379
BP	GO:0002363	alpha-beta T	1/9	19/18670	0.00912383	0.02521837	0.0084413
BP	GO:0045076	regulation of	1/9	19/18670	0.00912383	0.02521837	0.0084413
BP	GO:0050863	regulation of	2/9	314/18670	0.00938836	0.02521837	0.0084413
BP	GO:0032495	response to r	1/9	20/18670	0.00960198	0.02521837	0.0084413
BP	GO:0035458	cellular respo	1/9	20/18670	0.00960198	0.02521837	0.0084413
BP	GO:0043369	CD4-positive	1/9	20/18670	0.00960198	0.02521837	0.0084413
BP	GO:0045655	regulation of	1/9	20/18670	0.00960198	0.02521837	0.0084413
BP	GO:0032496	response to l	2/9	330/18670	0.0103296	0.02643904	0.00884989
BP	GO:0032656	regulation of	1/9	22/18670	0.01055765	0.02643904	0.00884989
BP	GO:0042094	interleukin-2	1/9	22/18670	0.01055765	0.02643904	0.00884989
BP	GO:0071359	cellular respo	1/9	22/18670	0.01055765	0.02643904	0.00884989
BP	GO:0007596	blood coagul	2/9	336/18670	0.01069313	0.02643904	0.00884989
BP	GO:0007599	hemostasis	2/9	341/18670	0.01100043	0.02643904	0.00884989
BP	GO:0032753	positive regu	1/9	23/18670	0.01103518	0.02643904	0.00884989
BP	GO:0036037	CD8-positive,	1/9	23/18670	0.01103518	0.02643904	0.00884989

BP	GO:0050817	coagulation	2/9	342/18670	0.01106236	0.02643904	0.00884989
BP	GO:0030098	lymphocyte d	2/9	353/18670	0.01175403	0.027814	0.00931013
BP	GO:0032616	interleukin-1	1/9	25/18670	0.01198963	0.02809334	0.00940363
BP	GO:0002460	adaptive imn	2/9	361/18670	0.01226897	0.02837622	0.00949832
BP	GO:0002360	T cell lineage	1/9	26/18670	0.01246654	0.02837622	0.00949832
BP	GO:0072539	T-helper 17 c	1/9	26/18670	0.01246654	0.02837622	0.00949832
BP	GO:0032673	regulation of	1/9	27/18670	0.01294325	0.02918337	0.00976849
BP	GO:0001773	myeloid denc	1/9	28/18670	0.01341976	0.02969743	0.00994056
BP	GO:0035456	response to i	1/9	28/18670	0.01341976	0.02969743	0.00994056
BP	GO:0033032	regulation of	1/9	29/18670	0.01389606	0.03019235	0.01010623
BP	GO:0072538	T-helper 17 t	1/9	29/18670	0.01389606	0.03019235	0.01010623
BP	GO:0032743	positive regu	1/9	30/18670	0.01437216	0.03066916	0.01026583
BP	GO:0046640	regulation of	1/9	30/18670	0.01437216	0.03066916	0.01026583
BP	GO:0045589	regulation of	1/9	32/18670	0.01532374	0.03241039	0.01084867
BP	GO:0032633	interleukin-4	1/9	33/18670	0.01579923	0.03283491	0.01099077
BP	GO:0046633	alpha-beta T	1/9	33/18670	0.01579923	0.03283491	0.01099077
BP	GO:0043368	positive T cel	1/9	34/18670	0.01627451	0.03324451	0.01112787
BP	GO:0045066	regulatory T	1/9	34/18670	0.01627451	0.03324451	0.01112787
BP	GO:0045622	regulation of	1/9	35/18670	0.01674958	0.033925	0.01135565
BP	GO:0030224	monocyte dif	1/9	36/18670	0.01722446	0.03402186	0.01138807
BP	GO:0032733	positive regu	1/9	36/18670	0.01722446	0.03402186	0.01138807
BP	GO:1903131	mononuclear	1/9	36/18670	0.01722446	0.03402186	0.01138807
BP	GO:0034122	negative regu	1/9	41/18670	0.01959576	0.03809369	0.01275103
BP	GO:0002683	negative regu	2/9	463/18670	0.01968384	0.03809369	0.01275103
BP	GO:0042110	T cell activati	2/9	464/18670	0.01976409	0.03809369	0.01275103
BP	GO:0097028	dendritic cell	1/9	42/18670	0.02006941	0.03837272	0.01284443
BP	GO:0043616	keratinocyte	1/9	43/18670	0.02054286	0.03896622	0.01304309
BP	GO:0051249	regulation of	2/9	485/18670	0.02148208	0.04012426	0.01343072
BP	GO:0045581	negative regu	1/9	45/18670	0.02148915	0.04012426	0.01343072
BP	GO:0032480	negative regu	1/9	46/18670	0.02196198	0.04031484	0.01349451
BP	GO:0043330	response to e	1/9	46/18670	0.02196198	0.04031484	0.01349451
BP	GO:0043370	regulation of	1/9	47/18670	0.02243462	0.04031484	0.01349451
BP	GO:0045058	T cell selectio	1/9	47/18670	0.02243462	0.04031484	0.01349451
BP	GO:2000107	negative regu	1/9	47/18670	0.02243462	0.04031484	0.01349451
BP	GO:0097300	programmed	1/9	49/18670	0.02337928	0.04169886	0.01395778
BP	GO:0032653	regulation of	1/9	52/18670	0.02479475	0.04389588	0.01469318
BP	GO:0043331	response to d	1/9	53/18670	0.02526616	0.04440157	0.01486245
BP	GO:0032663	regulation of	1/9	54/18670	0.02573738	0.04489951	0.01502913
BP	GO:0032613	interleukin-1	1/9	55/18670	0.02620839	0.04506335	0.01508397
BP	GO:0045620	negative regu	1/9	55/18670	0.02620839	0.04506335	0.01508397
BP	GO:0031663	lipopolysacch	1/9	58/18670	0.02762021	0.04681724	0.01567104
BP	GO:0042093	T-helper cell	1/9	58/18670	0.02762021	0.04681724	0.01567104
BP	GO:0043388	positive regu	1/9	59/18670	0.02809042	0.04719879	0.01579876
BP	GO:0002294	CD4-positive,	1/9	60/18670	0.02856042	0.04719879	0.01579876
BP	GO:0043966	histone H3 ac	1/9	60/18670	0.02856042	0.04719879	0.01579876
BP	GO:0002287	alpha-beta T	1/9	61/18670	0.02903022	0.04719879	0.01579876
BP	GO:0002293	alpha-beta T	1/9	61/18670	0.02903022	0.04719879	0.01579876
BP	GO:2000514	regulation of	1/9	61/18670	0.02903022	0.04719879	0.01579876
BP	GO:0032623	interleukin-2	1/9	62/18670	0.02949981	0.04731849	0.01583883
BP	GO:0070265	necrotic cell	1/9	62/18670	0.02949981	0.04731849	0.01583883

BP	GO:0046637	regulation of	1/9	63/18670	0.02996921	0.04775094	0.01598358
BP	GO:0032729	positive regu	1/9	65/18670	0.03090739	0.04891965	0.01637478
BP	GO:0043967	histone H4 ac	1/9	67/18670	0.03184477	0.05007171	0.0167604
BP	GO:0002292	T cell differer	1/9	68/18670	0.03231316	0.05047611	0.01689577
BP	GO:0061180	mammary gla	1/9	72/18670	0.03418469	0.05305286	0.01775828
BP	GO:0043367	CD4-positive,	1/9	74/18670	0.03511925	0.05415162	0.01812606
BP	GO:0071260	cellular respo	1/9	79/18670	0.03745213	0.05737859	0.01920622
BP	GO:2000106	regulation of	1/9	83/18670	0.03931483	0.05984868	0.02003303
BP	GO:0060021	roof of mout	1/9	89/18670	0.04210286	0.06368724	0.0213179
BP	GO:0035710	CD4-positive,	1/9	92/18670	0.04349418	0.06537804	0.02188386
BP	GO:0120162	positive regu	1/9	97/18670	0.04580905	0.06842727	0.02290453
BP	GO:0032649	regulation of	1/9	101/18670	0.04765736	0.07074602	0.02368068
BP	GO:1902106	negative regu	1/9	103/18670	0.04858033	0.07167097	0.02399028
BP	GO:0002286	T cell activati	1/9	106/18670	0.04996328	0.07325904	0.02452185
BP	GO:0050868	negative regu	1/9	112/18670	0.05272382	0.07683532	0.02571894
BP	GO:0032609	interferon-ga	1/9	113/18670	0.05318322	0.07703508	0.0257858
BP	GO:0002761	regulation of	1/9	117/18670	0.05501882	0.07921385	0.0265151
BP	GO:0051101	regulation of	1/9	124/18670	0.05822353	0.08332589	0.02789151
BP	GO:1903038	negative regu	1/9	129/18670	0.06050668	0.08607796	0.02881271
BP	GO:0050715	positive regu	1/9	139/18670	0.06505824	0.09200544	0.0307968
BP	GO:0030879	mammary gla	1/9	143/18670	0.06687336	0.09292287	0.03110389
BP	GO:0106106	cold-induced	1/9	143/18670	0.06687336	0.09292287	0.03110389
BP	GO:0120161	regulation of	1/9	143/18670	0.06687336	0.09292287	0.03110389
BP	GO:0051250	negative regu	1/9	146/18670	0.06823265	0.09426361	0.03155267
BP	GO:1990845	adaptive ther	1/9	154/18670	0.07184882	0.09823428	0.03288177
BP	GO:1903707	negative regu	1/9	155/18670	0.07229996	0.09823428	0.03288177
BP	GO:0016573	histone acety	1/9	156/18670	0.07275091	0.09823428	0.03288177
BP	GO:0042129	regulation of	1/9	156/18670	0.07275091	0.09823428	0.03288177
CC	GO:0000790	nuclear chror	2/9	377/19717	0.01201047	0.07747104	0.05436564
CC	GO:0000788	nuclear nucle	1/9	38/19717	0.01721579	0.07747104	0.05436564
MF	GO:0001228	DNA-binding	6/9	439/17697	1.7763E-08	7.1053E-08	3.7396E-08
MF	GO:0001227	DNA-binding	2/9	242/17697	0.00629272	0.01258544	0.00662391
MF	GO:0001158	enhancer sec	1/9	119/17697	0.0589292	0.06565491	0.03455522
MF	GO:0035326	enhancer bin	1/9	133/17697	0.06565491	0.06565491	0.03455522
KEGG	hsa05133	Pertussis	3/7	76/8076	2.7286E-05	0.00068214	0.00028722
KEGG	hsa04620	Toll-like rece	3/7	104/8076	6.9943E-05	0.00087429	0.00036812
KEGG	hsa05162	Measles	3/7	139/8076	0.00016603	0.0013836	0.00058257
KEGG	hsa05160	Hepatitis C	3/7	157/8076	0.00023823	0.00143261	0.0006032
KEGG	hsa05164	Influenza A	3/7	171/8076	0.00030668	0.00143261	0.0006032
KEGG	hsa04621	NOD-like rec	3/7	181/8076	0.00036268	0.00143261	0.0006032
KEGG	hsa05167	Kaposi sarcor	3/7	193/8076	0.00043817	0.00143261	0.0006032
KEGG	hsa05169	Epstein-Barr	3/7	202/8076	0.00050102	0.00143261	0.0006032
KEGG	hsa05203	Viral carcinog	3/7	204/8076	0.00051574	0.00143261	0.0006032
KEGG	hsa04623	Cytosolic DN	2/7	63/8076	0.00122648	0.0030662	0.00129103
KEGG	hsa04622	RIG-I-like rec	2/7	70/8076	0.00151222	0.00343687	0.0014471
KEGG	hsa05165	Human papill	3/7	331/8076	0.00211147	0.00439889	0.00185216
KEGG	hsa04625	C-type lectin	2/7	104/8076	0.0033069	0.00635942	0.00267765
KEGG	hsa05168	Herpes simpl	3/7	498/8076	0.00676581	0.01208181	0.00508708
KEGG	hsa05161	Hepatitis B	2/7	162/8076	0.00786022	0.01310037	0.00551594
KEGG	hsa04917	Prolactin sigr	1/7	70/8076	0.0591399	0.0924061	0.03890783

geneID	Count
IRF8/IRF1/IRI	9
IRF8/IRF1/IRI	9
IRF8/IRF1/IRI	9
IRF8/IRF1/IRI	9
IRF8/IRF1/IRI	9
IRF8/IRF1/IRI	9
IRF1/IRF2/IRI	6
IRF1/IRF3/IRI	4
IRF1/IRF2/IRI	6
IRF1/IRF3/IRI	4
IRF1/IRF3/IRI	4
IRF8/IRF1/IRI	6
IRF1/IRF3/IRI	4
IRF3/IRF5/IRI	3
IRF1/IRF3/IRI	4
IRF1/IRF3/IRI	4
IRF1/IRF3/IRI	4
IRF3/IRF5/IRI	3
IRF1/IRF3/IRI	4
IRF3/IRF5/IRI	3
IRF1/IRF3/IRI	4
IRF8/IRF1/IRI	3
IRF1/IRF3/IRI	4
IRF8/IRF1/IRI	3
IRF8/IRF1/IRI	3
IRF1/IRF4/IRI	3
IRF1/IRF3/IRI	4
IRF3/IRF7	2
IRF1/IRF3/IRI	4
IRF3/IRF7	2
IRF3/IRF7	2
IRF3/IRF7	2
IRF1/IRF3/IRI	4
IRF1/IRF3/IRI	4
IRF3/IRF7	2
IRF8/IRF4	2
IRF8/IRF4	2
IRF3/IRF7	2
IRF3/IRF7	2
IRF3/IRF7	2
IRF3/IRF7	2
IRF3/IRF7	2
IRF1/IRF7	2
IRF3/IRF7	2
IRF1/IRF4/IRI	3
IRF3/IRF7	2
IRF3/IRF7	2
IRF3/IRF7	2
IRF3/IRF7	2

IRF1/IRF4	2
IRF8/IRF3/IRI	3
IRF8/IRF4/IRI	3
IRF1/IRF4	2
IRF1/IRF4	2
IRF3/IRF7	2
IRF1/IRF4/IRI	3
IRF1/IRF4	2
IRF1/IRF4	2
IRF1/IRF4	2
IRF1/IRF7	2
IRF1/IRF4	2
IRF3/IRF7	2
IRF3/IRF7	2
IRF4/IRF7	2
IRF8/IRF3	2
IRF8/IRF3	2
IRF7	1
IRF7	1
IRF5	1
IRF8/IRF3	2
IRF1/IRF6	2
IRF1/IRF4	2
IRF4	1
IRF4	1
IRF1	1
IRF4	1
IRF1	1
IRF3/IRF7	2
IRF4	1
IRF7	1
IRF4	1
IRF6	1
IRF3	1
IRF4	1
IRF4	1
IRF4	1
IRF1/IRF4	2
IRF5	1
IRF1	1
IRF4	1
IRF7	1
IRF8/IRF3	2
IRF4	1
IRF4	1
IRF3	1
IRF1/IRF2	2
IRF1/IRF2	2
IRF4	1
IRF1	1

IRF1/IRF2	2
IRF1/IRF4	2
IRF4	1
IRF4/IRF7	2
IRF4	1
IRF4	1
IRF4	1
IRF4	1
IRF1	1
IRF7	1
IRF4	1
IRF4	1
IRF1	1
IRF1	1
IRF4	1
IRF1	1
IRF4	1
IRF1	1
IRF4	1
IRF7	1
IRF4	1
IRF7	1
IRF4	1
IRF1/IRF4	2
IRF1/IRF4	2
IRF4	1
IRF6	1
IRF1/IRF4	2
IRF1	1
IRF3	1
IRF3	1
IRF4	1
IRF4	1
IRF7	1
IRF3	1
IRF4	1
IRF3	1
IRF4	1
IRF4	1
IRF1	1
IRF3	1
IRF4	1
IRF4	1
IRF4	1
IRF4	1
IRF4	1
IRF4	1
IRF4	1
IRF4	1
IRF3	1

IRF4	1
IRF8	1
IRF4	1
IRF4	1
IRF6	1
IRF4	1
IRF1	1
IRF7	1
IRF6	1
IRF4	1
IRF4	1
IRF8	1
IRF1	1
IRF4	1
IRF1	1
IRF8	1
IRF7	1
IRF4	1
IRF1	1
IRF3	1
IRF6	1
IRF4	1
IRF4	1
IRF1	1
IRF4	1
IRF1	1
IRF4	1
IRF1	1
IRF1/IRF4	2
IRF4	1
IRF1/IRF2/IRI	6
IRF8/IRF3	2
IRF7	1
IRF7	1
IRF8/IRF1/IRI	3
IRF3/IRF5/IRI	3
IRF3/IRF7/IRI	3
IRF3/IRF7/IRI	3
IRF3/IRF7/IRI	3
IRF3/IRF7/IRI	3
IRF3/IRF7/IRI	3
IRF3/IRF7/IRI	3
IRF3/IRF7/IRI	3
IRF3/IRF7	2
IRF3/IRF7	2
IRF1/IRF3/IRI	3
IRF1/IRF9	2
IRF3/IRF7/IRI	3
IRF3/IRF7	2
IRF1	1
