# Supplementary material for: A Comprehensive Analysis of Interferon Regulatory Factor Expression: Correlation with Immune Cell Infiltration and Patient Prognosis in Endometrial Carcinoma
Source: Biomed Res Int. 2022 Aug 8;2022:7948898. doi: 10.1155/2022/7948898 (PMC9381850; doi:10.1155/2022/7948898)
Supplement: Supplementary 4 — Supplementary Table 4: the relationship between IRFs and immune cell infiltration. [file 7948898.f4.pdf]

id	Immune cells	Pearson	P(Pearson)	Spearman	P(Spearman)
IRF1	aDC	0.610	<0.001	0.586	<0.001
IRF1	B cells	0.460	<0.001	0.457	<0.001
IRF1	CD8 T cells	0.325	<0.001	0.314	<0.001
IRF1	Cytotoxic cells	0.530	<0.001	0.506	<0.001
IRF1	DC	0.311	<0.001	0.281	<0.001
IRF1	Eosinophils	0.151	<0.001	0.130	0.002
IRF1	iDC	0.341	<0.001	0.356	<0.001
IRF1	Macrophages	0.353	<0.001	0.328	<0.001
IRF1	Mast cells	0.094	0.027	0.089	0.036
IRF1	Neutrophils	0.352	<0.001	0.376	<0.001
IRF1	NK CD56bright ce	0.172	<0.001	0.150	<0.001
IRF1	NK CD56dim cells	0.388	<0.001	0.356	<0.001
IRF1	NK cells	-0.082	0.056	-0.094	0.028
IRF1	pDC	0.048	0.260	0.062	0.149
IRF1	T cells	0.525	<0.001	0.489	<0.001
IRF1	T helper cells	0.218	<0.001	0.187	<0.001
IRF1	Tcm	0.147	<0.001	0.157	<0.001
IRF1	Tem	0.223	<0.001	0.182	<0.001
IRF1	TFH	0.316	<0.001	0.289	<0.001
IRF1	Tgd	0.115	0.007	0.082	0.054
IRF1	Th1 cells	0.551	<0.001	0.502	<0.001
IRF1	Th17 cells	0.079	0.064	0.068	0.109
IRF1	Th2 cells	0.118	0.006	0.110	0.010
IRF1	TReg	0.323	<0.001	0.313	<0.001
IRF2	aDC	0.218	<0.001	0.216	<0.001
IRF2	B cells	0.080	0.059	0.089	0.036
IRF2	CD8 T cells	0.089	0.038	0.067	0.117
IRF2	Cytotoxic cells	0.065	0.129	0.093	0.028
IRF2	DC	0.005	0.898	0.011	0.794
IRF2	Eosinophils	0.184	<0.001	0.189	<0.001
IRF2	iDC	0.178	<0.001	0.188	<0.001
IRF2	Macrophages	0.168	<0.001	0.149	<0.001
IRF2	Mast cells	0.031	0.463	0.071	0.094
IRF2	Neutrophils	0.163	<0.001	0.213	<0.001
IRF2	NK CD56bright ce	0.038	0.377	0.068	0.108
IRF2	NK CD56dim cells	0.014	0.742	0.054	0.208
IRF2	NK cells	-0.066	0.119	-0.079	0.064
IRF2	pDC	-0.309	<0.001	-0.247	<0.001
IRF2	T cells	0.167	<0.001	0.169	<0.001
IRF2	T helper cells	0.331	<0.001	0.236	<0.001
IRF2	Tcm	0.336	<0.001	0.416	<0.001
IRF2	Tem	0.053	0.216	0.072	0.089
IRF2	TFH	0.089	0.036	0.081	0.058
IRF2	Tgd	0.087	0.040	0.067	0.114
IRF2	Th1 cells	0.152	<0.001	0.160	<0.001
IRF2	Th17 cells	0.032	0.456	0.051	0.233
IRF2	Th2 cells	0.148	<0.001	0.110	0.010
IRF2	TReg	0.041	0.337	0.044	0.306
IRF3	aDC	0.200	<0.001	0.183	<0.001
IRF3	B cells	0.113	0.008	0.072	0.091
IRF3	CD8 T cells	0.250	<0.001	0.133	0.002
IRF3	Cytotoxic cells	0.162	<0.001	0.092	0.031
IRF3	DC	0.096	0.024	0.049	0.247
IRF3	Eosinophils	-0.085	0.046	-0.171	<0.001
IRF3	iDC	-0.092	0.031	-0.189	<0.001
IRF3	Macrophages	-0.039	0.363	-0.028	0.508
IRF3	Mast cells	-0.047	0.274	-0.113	0.008

IRF3	Neutrophils	-0.115	0.007	-0.154	<0.001
IRF3	NK CD56bright ce	0.066	0.122	-0.054	0.208
IRF3	NK CD56dim cells	0.156	<0.001	0.070	0.102
IRF3	NK cells	0.129	0.002	-0.059	0.168
IRF3	pDC	0.117	0.006	0.076	0.075
IRF3	T cells	0.060	0.156	0.030	0.480
IRF3	T helper cells	-0.124	0.003	-0.044	0.303
IRF3	Tcm	-0.260	<0.001	-0.155	<0.001
IRF3	Tem	0.039	0.360	0.015	0.725
IRF3	TFH	0.110	0.010	0.073	0.086
IRF3	Tgd	0.026	0.548	0.019	0.653
IRF3	Th1 cells	0.194	<0.001	0.147	<0.001
IRF3	Th17 cells	-0.024	0.569	-0.052	0.224
IRF3	Th2 cells	0.110	0.010	0.165	<0.001
IRF3	TReg	0.099	0.020	0.061	0.151
IRF4	aDC	0.408	<0.001	0.381	<0.001
IRF4	B cells	0.713	<0.001	0.717	<0.001
IRF4	CD8 T cells	0.410	<0.001	0.478	<0.001
IRF4	Cytotoxic cells	0.560	<0.001	0.599	<0.001
IRF4	DC	0.469	<0.001	0.476	<0.001
IRF4	Eosinophils	0.146	<0.001	0.180	<0.001
IRF4	iDC	0.330	<0.001	0.398	<0.001
IRF4	Macrophages	0.416	<0.001	0.442	<0.001
IRF4	Mast cells	0.168	<0.001	0.210	<0.001
IRF4	Neutrophils	0.314	<0.001	0.379	<0.001
IRF4	NK CD56bright ce	-0.035	0.409	0.001	0.981
IRF4	NK CD56dim cells	0.478	<0.001	0.512	<0.001
IRF4	NK cells	-0.176	<0.001	-0.107	0.012
IRF4	pDC	0.230	<0.001	0.208	<0.001
IRF4	T cells	0.692	<0.001	0.748	<0.001
IRF4	T helper cells	0.485	<0.001	0.525	<0.001
IRF4	Tcm	0.259	<0.001	0.269	<0.001
IRF4	Tem	0.490	<0.001	0.459	<0.001
IRF4	TFH	0.517	<0.001	0.522	<0.001
IRF4	Tgd	0.236	<0.001	0.224	<0.001
IRF4	Th1 cells	0.608	<0.001	0.608	<0.001
IRF4	Th17 cells	0.089	0.036	0.101	0.018
IRF4	Th2 cells	0.242	<0.001	0.270	<0.001
IRF4	TReg	0.493	<0.001	0.506	<0.001
IRF5	aDC	0.380	<0.001	0.372	<0.001
IRF5	B cells	0.332	<0.001	0.331	<0.001
IRF5	CD8 T cells	0.090	0.034	0.069	0.108
IRF5	Cytotoxic cells	0.242	<0.001	0.227	<0.001
IRF5	DC	0.120	0.005	0.112	0.009
IRF5	Eosinophils	0.083	0.051	0.052	0.225
IRF5	iDC	0.134	0.002	0.131	0.002
IRF5	Macrophages	0.171	<0.001	0.164	<0.001
IRF5	Mast cells	-0.045	0.291	-0.071	0.094
IRF5	Neutrophils	0.170	<0.001	0.162	<0.001
IRF5	NK CD56bright ce	0.089	0.036	0.068	0.112
IRF5	NK CD56dim cells	0.097	0.023	0.065	0.124
IRF5	NK cells	-0.116	0.006	-0.147	<0.001
IRF5	pDC	-0.011	0.804	-0.016	0.712
IRF5	T cells	0.176	<0.001	0.159	<0.001
IRF5	T helper cells	-0.026	0.538	-0.038	0.377
IRF5	Tcm	-0.016	0.706	0.003	0.940
IRF5	Tem	0.065	0.126	0.081	0.058
IRF5	TFH	0.077	0.071	0.064	0.132

IRF5	Tgd	-0.044	0.300	-0.047	0.271
IRF5	Th1 cells	0.206	<0.001	0.205	<0.001
IRF5	Th17 cells	0.147	<0.001	0.146	<0.001
IRF5	Th2 cells	-0.061	0.152	-0.063	0.137
IRF5	TReg	0.104	0.015	0.105	0.014
IRF6	aDC	-0.145	<0.001	-0.150	<0.001
IRF6	B cells	-0.201	<0.001	-0.206	<0.001
IRF6	CD8 T cells	-0.182	<0.001	-0.183	<0.001
IRF6	Cytotoxic cells	-0.252	<0.001	-0.250	<0.001
IRF6	DC	-0.212	<0.001	-0.201	<0.001
IRF6	Eosinophils	0.133	0.002	0.128	0.003
IRF6	iDC	0.001	0.975	0.000	0.996
IRF6	Macrophages	-0.123	0.004	-0.100	0.019
IRF6	Mast cells	0.136	0.001	0.098	0.021
IRF6	Neutrophils	0.028	0.505	0.014	0.736
IRF6	NK CD56bright ce	0.031	0.461	-0.003	0.936
IRF6	NK CD56dim cells	-0.266	<0.001	-0.258	<0.001
IRF6	NK cells	0.045	0.287	0.000	0.992
IRF6	pDC	-0.282	<0.001	-0.329	<0.001
IRF6	T cells	-0.165	<0.001	-0.148	<0.001
IRF6	T helper cells	-0.007	0.869	0.011	0.792
IRF6	Tcm	0.204	<0.001	0.281	<0.001
IRF6	Tem	-0.216	<0.001	-0.152	<0.001
IRF6	TFH	-0.153	<0.001	-0.155	<0.001
IRF6	Tgd	-0.122	0.004	-0.066	0.122
IRF6	Th1 cells	-0.245	<0.001	-0.197	<0.001
IRF6	Th17 cells	0.085	0.047	0.013	0.761
IRF6	Th2 cells	-0.070	0.102	-0.023	0.584
IRF6	TReg	-0.234	<0.001	-0.241	<0.001
IRF7	aDC	0.548	<0.001	0.549	<0.001
IRF7	B cells	0.210	<0.001	0.224	<0.001
IRF7	CD8 T cells	0.172	<0.001	0.115	0.007
IRF7	Cytotoxic cells	0.257	<0.001	0.241	<0.001
IRF7	DC	0.087	0.041	0.072	0.091
IRF7	Eosinophils	0.023	0.586	-0.048	0.263
IRF7	iDC	0.035	0.414	0.011	0.799
IRF7	Macrophages	0.028	0.511	0.050	0.240
IRF7	Mast cells	-0.016	0.712	-0.042	0.327
IRF7	Neutrophils	0.043	0.311	0.051	0.230
IRF7	NK CD56bright ce	0.167	<0.001	0.104	0.015
IRF7	NK CD56dim cells	0.127	0.003	0.096	0.024
IRF7	NK cells	0.195	<0.001	0.095	0.026
IRF7	pDC	0.125	0.003	0.091	0.032
IRF7	T cells	0.133	0.002	0.137	0.001
IRF7	T helper cells	-0.122	0.004	-0.103	0.016
IRF7	Tcm	-0.285	<0.001	-0.212	<0.001
IRF7	Tem	0.017	0.689	0.009	0.824
IRF7	TFH	0.134	0.002	0.129	0.002
IRF7	Tgd	-0.062	0.145	-0.066	0.120
IRF7	Th1 cells	0.191	<0.001	0.183	<0.001
IRF7	Th17 cells	0.020	0.643	0.010	0.817
IRF7	Th2 cells	-0.061	0.155	-0.040	0.346
IRF7	TReg	0.244	<0.001	0.228	<0.001
IRF8	aDC	0.387	<0.001	0.383	<0.001
IRF8	B cells	0.464	<0.001	0.475	<0.001
IRF8	CD8 T cells	0.233	<0.001	0.274	<0.001
IRF8	Cytotoxic cells	0.446	<0.001	0.467	<0.001
IRF8	DC	0.325	<0.001	0.317	<0.001

IRF8	Eosinophils	0.225	<0.001	0.221	<0.001
IRF8	iDC	0.413	<0.001	0.446	<0.001
IRF8	Macrophages	0.500	<0.001	0.511	<0.001
IRF8	Mast cells	0.120	0.005	0.125	0.003
IRF8	Neutrophils	0.322	<0.001	0.352	<0.001
IRF8	NK CD56bright ce	0.007	0.873	0.034	0.422
IRF8	NK CD56dim cells	0.358	<0.001	0.348	<0.001
IRF8	NK cells	-0.109	0.010	-0.069	0.107
IRF8	pDC	0.062	0.145	0.063	0.138
IRF8	T cells	0.536	<0.001	0.548	<0.001
IRF8	T helper cells	0.328	<0.001	0.330	<0.001
IRF8	Tcm	0.267	<0.001	0.271	<0.001
IRF8	Tem	0.349	<0.001	0.320	<0.001
IRF8	TFH	0.361	<0.001	0.380	<0.001
IRF8	Tgd	0.204	<0.001	0.193	<0.001
IRF8	Th1 cells	0.515	<0.001	0.499	<0.001
IRF8	Th17 cells	0.084	0.049	0.102	0.017
IRF8	Th2 cells	0.205	<0.001	0.196	<0.001
IRF8	TReg	0.317	<0.001	0.336	<0.001
IRF9	aDC	0.391	<0.001	0.393	<0.001
IRF9	B cells	0.117	0.006	0.134	0.002
IRF9	CD8 T cells	0.030	0.478	0.045	0.288
IRF9	Cytotoxic cells	0.020	0.636	0.042	0.325
IRF9	DC	-0.154	<0.001	-0.143	<0.001
IRF9	Eosinophils	0.096	0.025	0.037	0.383
IRF9	iDC	-0.132	0.002	-0.125	0.003
IRF9	Macrophages	0.082	0.054	0.060	0.160
IRF9	Mast cells	-0.006	0.879	-0.012	0.779
IRF9	Neutrophils	-0.050	0.238	-0.051	0.236
IRF9	NK CD56bright ce	-0.072	0.089	-0.091	0.034
IRF9	NK CD56dim cells	-0.108	0.011	-0.118	0.005
IRF9	NK cells	-0.092	0.031	-0.089	0.036
IRF9	pDC	-0.188	<0.001	-0.165	<0.001
IRF9	T cells	0.017	0.694	0.015	0.719
IRF9	T helper cells	0.172	<0.001	0.143	<0.001
IRF9	Tcm	0.170	<0.001	0.192	<0.001
IRF9	Tem	0.019	0.659	0.028	0.513
IRF9	TFH	0.034	0.424	0.055	0.193
IRF9	Tgd	-0.055	0.196	-0.056	0.189
IRF9	Th1 cells	0.115	0.007	0.121	0.005
IRF9	Th17 cells	-0.011	0.788	-0.013	0.754
IRF9	Th2 cells	-0.044	0.299	-0.037	0.389
IRF9	TReg	-0.003	0.944	0.015	0.732
